# Creativity and Leisure During COVID-19: Examining the Relationship Between Leisure Activities, Motivations, and Psychological Well-Being

**DOI:** 10.3389/fpsyg.2021.609967

**Published:** 2021-07-05

**Authors:** K. F. Morse, Philip A. Fine, Kathryn J. Friedlander

**Affiliations:** School of Psychology and Wellbeing, University of Buckingham, Buckingham, United Kingdom

**Keywords:** COVID-19, creativity, hobbies, leisure activities, leisure motivations, leisure disengagement, psychological well-being, RIASEC

## Abstract

Social distancing policies have been implemented around the world to reduce the spread of coronavirus disease 2019 (COVID-19). These measures have included temporary restrictions on mass gatherings and the closure of public facilities, limiting the pursuit of leisure activities such as travel while allowing more time for at-home pursuits, including creative activities such as gardening and painting. Previous research has demonstrated the benefits of physical activity for psychological well-being during COVID-19, but less attention has been given to the potential benefits of creative pursuits, such as arts and music. The present study investigated changes in the pursuit of creative, non-creative, and physical leisure activities and the relationship between engaging in leisure, the motivations for and barriers to pursuing these activities, and psychological well-being during COVID-19. A total of 3,827 participants from 74 countries completed an online leisure activities questionnaire and the World Health Organization Five Well-Being Index. Logistic regression indicated that gender, age, social distancing adherence, and employment status significantly predicted leisure engagement during COVID-19. Compared to sports and outdoor pursuits, participation in creative activities was generally more likely to increase during this period, while participation in non-creative activities was less likely to increase. Multiple linear regression indicated that maintaining or increasing time on leisure activities significantly predicted well-being during COVID-19, with increased time spent on home crafts and artisanship, fine arts, musical and performing arts engagement, sports and outdoor pursuits, niche and IT interests, and language activities each predicting higher well-being outcomes. Motivations such as seeking creative expression and mental stimulation, keeping fit, and maintaining social connections also predicted higher well-being. These findings suggest that participation in both physical and creative leisure activities may offer protective benefits for well-being during COVID-19, and that strategies to promote engagement in creative activities should also be considered in future guidance for mental health during periods of lockdown or isolation.

## Introduction

The COVID-19 pandemic has altered many aspects of daily life, including the pursuit of leisure activities. Since January 2020, nearly all countries affected by COVID-19 have implemented social distancing measures to mitigate spread of the virus (Chinazzi et al., 2020). Social distancing refers to measures taken to reduce social interaction and thus the spread of COVID-19, including behaviors such as: maintaining spatial distance from strangers and household members exhibiting symptoms; avoiding non-essential travel, use of public transport, and social gatherings; and working from home when possible (e.g., United Kingdom Cabinet Office, 2021). Subsequently, many employers, businesses, and public facilities have temporarily closed or shifted to remote operations, and individuals have been asked to remain at home under lockdown or other stay-at-home orders (Sen-Crowe et al., 2020). Lifestyle changes due to working from home and reduced commuting hours have left many with additional free time for leisure, that is, any activity voluntarily undertaken during free time, such as painting or exercise (Paggi et al., 2016; Office of National Statistics, 2020). However, increased time for leisure has coincided with restricted access to popular leisure facilities such as gyms and restaurants (Courtemanche et al., 2020), causing many to look toward activities available closer to home. In some cases, this may result in activities being dropped and substituted for comparable alternatives, e.g., outdoor running instead of gym-going or watching concerts online instead of in person, whereas in other cases this may facilitate the pursuit of an entirely new hobby. Importantly, the range of possible activities that an individual might undertake is in part limited by regional social distancing guidance and/or access to various facilities.

Studies on the psychosocial effects of COVID-19 have highlighted an increase in mental health problems such as depression and anxiety (Qiu et al., 2020; Torales et al., 2020; Williams et al., 2020), and engaging in leisure activities may provide protective benefits for psychological well-being (Kuykendall et al., 2018). The National Health Service (2020), World Health Organization (2020a), Mayo Clinic (2020), and other leading health organizations have emphasized the importance of allocating time for hobbies and leisure under their COVID-19 mental health support strategies. However, research has primarily focused on the benefits of engaging in physical leisure during COVID-19, such as walking or running, as well as gardening and other outdoor pursuits (Carriedo et al., 2020; Chtourou et al., 2020; Lades et al., 2020). Increased interest in running and home workouts has also received extensive coverage from news and popular media outlets since the outbreak of the global pandemic (Bryant, 2020; Lufkin, 2020; Minsberg, 2020). This guidance is typically in line with general health advice on the benefits of physical activity independently of the global pandemic (World Health Organization, 2020b).

On the other hand, considerably less attention has been given to the potential psychological benefits of participating in creative activities during COVID-19. For the purposes of the present study, a creative activity is considered any activity that involves the production of novel ideas or “expressing oneself in an original and useful way” (Conner et al., 2016 p. 3). These activities include, for example, traditional artistic pursuits such as painting and amateur music-making, as well as less commonly regarded creative activities such as baking, knitting, and mentally stimulating creative activities including programming, language learning, and writing. Although not the conventional representation of “creative” fine arts activities, these latter pursuits facilitate novel creative expression, and may be likened to the distinction between higher-level “professional” and personal or “little” creative pursuits outlined in the Four C Model of Creativity (Kaufman and Beghetto, 2009). To date, analyses of web search trends and commentary articles have highlighted increased public interest in cooking and baking (Goldman, 2020; Public Health Agency, 2020), jigsaw puzzles (McNeely, 2020), and a range of artistic pursuits (Willis, 2020) during the COVID-19 pandemic, but empirical research is currently lacking (Kiraly et al., 2020). The present study sought to characterize changes in various domains of leisure during COVID-19 (e.g., arts, exercise), and the relationship between increased participation in leisure, motivations for and barriers to pursuing activities, and psychological well-being. Specific attention was given to how participation in creative activities compared to participation in non-creative and physical and outdoor pursuits. First, a brief review of the literature on well-being during COVID-19, leisure and well-being, and the interplay between leisure and well-being during COVID-19 follows below.

To date, research has suggested primarily negative effects of COVID-19 on mental health and well-being, consistent with reports on psychological health during past pandemics such as SARS-CoV-1 and Ebola (Lau et al., 2006; Jalloh et al., 2018; Brooks et al., 2020; Matias et al., 2020; Rajkumar, 2020). For example, in an analysis of well-being in the United Kingdom (UK) during COVID-19, Banks and Xu (2020) reported an average 8.1% decrease in mental health indicators, with the largest decreases observed among young women. Studies have also reported increased incidence of depression and anxiety (Qiu et al., 2020), health anxiety and financial worry (Tull et al., 2020), and self-harm behaviors (Iob et al., 2020) associated with the pandemic. However, a study by Foa et al. (2020) using measures such as the UK “YouGov” mood tracker and web search trends in English-speaking countries suggested negative emotions rose sharply at the beginning of the pandemic but fell after the implementation of lockdown measures, although effects were differentially experienced across social and economic groups. These variant findings suggest that more research is needed on well-being during the COVID-19 pandemic, and particularly on measures to promote mental health during lockdown or isolation. Long-standing evidence indicates a strong link between participation in leisure activities and well-being (Kuykendall et al., 2018), including both fitness-related and creative pursuits, and investigating engagement in these activities during COVID-19 may offer informative insights for the promotion of mental health.

Broadly speaking, participation in leisure activities is strongly related to well-being across the lifespan (Adams et al., 2011; Leversen et al., 2012; Ryu and Heo, 2018), and this relationship may have important implications for mental health during COVID-19. For example, extensive meta-analytic review has supported the relationship between physical leisure and well-being (Wiese et al., 2018; Buecker et al., 2020). However, engaging in creative pursuits such as arts (Wheatley and Bickerton, 2019) and music (Fritz and Avsec, 2007) may also offer benefits for psychological health. Generally, the association between well-being and leisure is considered to be reciprocal (i.e., leisure enhances well-being and well-being affects the choice or ability to pursue leisure), but dominant psychological perspectives posit a primarily bottom-up approach through which *subjectively valued* leisure exerts direct influence on psychological well-being (Newman et al., 2014). These theories make a critical distinction between *leisure engagement* (i.e., participating in or having time for leisure) and *leisure satisfaction* (i.e., the perceived value of specific leisure activities) (Kuykendall et al., 2018). Although leisure engagement is considered precursory to satisfaction, the latter is ultimately thought to influence psychological well-being.

Specifically, Newman et al. (2014) propose that five psychological mechanisms mediate the relationship between leisure and well-being: work detachment-recovery, autonomous motivation, mastery and skill development, meaning-making, and social affiliation (known as the “DRAMMA” model). This perspective is supported by empirical and meta-analytic findings (Schimmack, 2008; Kuykendall et al., 2015), and suggests that leisure activities are valued when they promote well-being through at least one of these mechanisms. For example, previous research on the motivations for pursuing creative activities suggests that the strongest motivation is enjoyment, though certain activities have also been linked to self-expression and prosocial motives (Benedek et al., 2019). In the context of COVID-19, the DRAMMA mechanisms may correspond with the motivations for pursuing leisure activities. For example, increasing time on activities to cope with anxiety may not be enough to reduce stress related to the pandemic (Balhara et al., 2020; Duncan et al., 2020). Similarly, differences in well-being might be anticipated between those who participated in leisure to occupy additional free time and those motivated by intrinsic reasons (e.g., developing a new skill or seeking creative expression), with the pursuit of valued leisure acting through psychological mechanisms to enhance well-being. To this end, the present study investigated both changes in leisure engagement as well as the underlying psychological motivations, with particular attention given to the impact of motivations such as seeking creative expression and mental stimulation.

Taken together, the study of leisure activities and well-being during COVID-19 has been approached from several perspectives, namely: (1) identifying the populations that have experienced increased time available for leisure; (2) determining the activities on which added leisure time has been spent; and (3) identifying the protective or maladaptive characteristics of these activities. In the first instance, increased availability of free time during COVID-19 has been disproportionately observed across the population. While many individuals working from home have experienced more time available for leisure, frontline workers and those with additional caring responsibilities may have found their free time more limited: A survey by the UK Office of National Statistics (ONS) found that low-income households reported less free time available during COVID-19 due to undertaking additional paid working hours, accentuating that increased time for leisure is not a universal experience (Office of National Statistics, 2020). Additionally, the experience of working from home has had variable effects on stress and productivity, allowing more leisure time for some and less for others (Ford et al., 2020). Similarly, research has also revealed differences in the pursuit of physical activity during COVID-19 based on gender, age, and annual household income (Smith et al., 2020). The current study therefore considered perspectives from both those who increased and those who decreased time on leisure activities during COVID-19, looking at motivations for leisure among the former and barriers to leisure among the latter.

The Office of National Statistics (2020) survey also found that, on average across the population, British adults spent ~1 hour less on transport and 44 min more on leisure activities daily during lockdown, with gardening, do-it-yourself (DIY) activities, resting, video chatting, and online streaming experiencing the greatest average increase compared to 2014–15. Similar findings reported by *The New York Times* market research revealed that since the start of COVID-19, Americans have increased their monetary expenditure on home improvement, gaming, and video streaming, while decreasing spending on movie theaters, travel, and fitness (among other activities; Leatherby and Gelles, 2020). However, both studies focused on overall changes in leisure engagement at the population level (i.e., activities that increased or decreased), but not the corresponding psychological motivations or their relationship to individual well-being. Moreover, these studies generally explored a limited range of leisure activities (e.g., socializing, media use, and outdoor activities) but did not extend to creative pursuits.

Empirical research has reported similar findings. For example, a study on behavior during lockdown in Spain reported engagement in telephone and video calls (in ~97% of responses), social media usage (85%), streaming or watching TV (79%), reading (52%), and physical exercise (49%) (Rodriguez-Rey et al., 2020). Engaging in leisure during the preceding 24 hours was related to lower psychological stress, anxiety, and depression. Similarly, in Ireland, Lades et al. (2020) reported that outdoor activities (going for walks, gardening, and exercise), engaging in hobbies, and childcare were associated with positive emotions (happiness, relaxation, and energy levels) using a day-reconstruction method.

To date, an abundance of research has emphasized the benefits of physical leisure during COVID-19, such as walking or running (Carriedo et al., 2020; Chtourou et al., 2020; Lesser and Nienhuis, 2020; Maugeri et al., 2020; Slimani et al., 2020). These findings are consistent with reports from the SARS-CoV-1 outbreak, in which ~35–40% of respondents reported more time spent on rest, relaxation, or exercise, and participation in these activities was associated with decreased stress perceptions (Lau et al., 2006). However, others have drawn attention to the potentially negative effects of certain activities including excessive internet and pornography use (Mestre-Bach et al., 2020) and gaming addiction (King et al., 2020; Ko and Yen, 2020), although exercise-based gaming during COVID-19 may have benefits for mental health (Viana and de Lira, 2020).

On the other hand, relatively little attention has been paid to the potential psychological effects of engaging in creative leisure activities during COVID-19, and the few studies conducted have focused primarily on behaviors in specific populations. For example, musical therapy interventions in Italy were shown to improve the well-being of staff working with COVID-19 patients (Giordano et al., 2020). Similarly, engaging in creative writing activities during lockdown in the United Kingdom was related to higher well-being in children and adolescents (Clark et al., 2020). The potential benefits of dance (Chtourou et al., 2020) and art therapy (Braus and Morton, 2020; Carr, 2020) have also been discussed. However, very little research has investigated the pursuit of creative leisure activities during COVID-19 among adults in home settings. Moreover, these studies generally focused on engagement in conventional creative activities (e.g., fine arts), but not every day personal expressions of “little” creativity in the form of activities such as baking or gardening (Kaufman and Beghetto, 2009). Thus, while most findings suggest positive effects of physical leisure on mental health during COVID-19, more research is needed on creative leisure activities, the motivations for pursuing leisure, and the association between leisure and well-being during COVID-19.

The present study sought to extend research on leisure and well-being during COVID-19 using a large-scale international survey comprising 3,827 participants. While the studies reviewed above primarily investigated leisure activities during the early stages of COVID-19 lockdown (March or April 2020 in most areas), the present research was conducted in late May to early June 2020, and therefore inquired about activities pursued during an extended COVID-19 period (compared to, e.g., the preceding 24 h, as is more commonly explored), with particular attention given to the pursuit of creative activities. The following questions were investigated:

(1) Were participant demographic characteristics (e.g., age, gender, and employment status) associated with increased time on leisure activities during COVID-19?
(2) Was increased time on activities during COVID-19 more likely in certain domains of leisure (e.g., creative arts, music) than others (e.g., sports and outdoor pursuits)? Were creative activities generally more likely to be taken up than non-creative activities?
(3) Did (i) increasing time on leisure and (ii) taking up new leisure activities predict well-being over and above potential confounding variables (e.g., age, gender, and employment status)?
(4) Was increased participation in certain domains of leisure (e.g., arts, music, and sports) predictive of well-being? How did the relationship between well-being and creative activities compare to that with non-creative activities?
(5) What were the overarching (i) motivations for and (ii) barriers to leisure during COVID-19, and were these factors predictive of well-being?

The operationalization of these questions is further described in the Materials and Methods section below.

## Materials and Methods

### Participants

A total of 5,263 participants were recruited through email, social media (e.g., Facebook, Twitter, and Reddit), and online referrals. The survey was advertised as “a study about keeping busy during COVID-19” and participants were invited to share their experiences of leisure time during the pandemic. Due to the nature of survey research, the sample was likely affected by self-selection bias, i.e., completed by those with the time or inclination to do so. Thus, the primary data analyses investigate leisure experiences among those who increased or maintained the same amount of time spent on activities during the pandemic, taking into account that this is not necessarily representative of the entire population. Barriers to leisure are also explored in a smaller sub-analysis (see Barriers to Leisure).

Before starting the survey, all participants provided informed consent and confirmed that they satisfied the two following inclusion criteria: (1) they were at least 16 years of age; and (2) they were living in an area that was or had previously been affected by COVID-19. Participants below 16 years of age or who indicated that they were not living in an area affected by COVID-19 were unable to proceed to the survey. No other restrictions were made on place of residence or other demographic characteristics. Because demographic characteristics were not known prior to recruitment, they are described in the first section of the Results. All responses were anonymous and no monetary compensation was offered. The study received full ethical approval from the School of Psychology and Wellbeing Ethics Committee, University of Buckingham.

**Figure 1:**
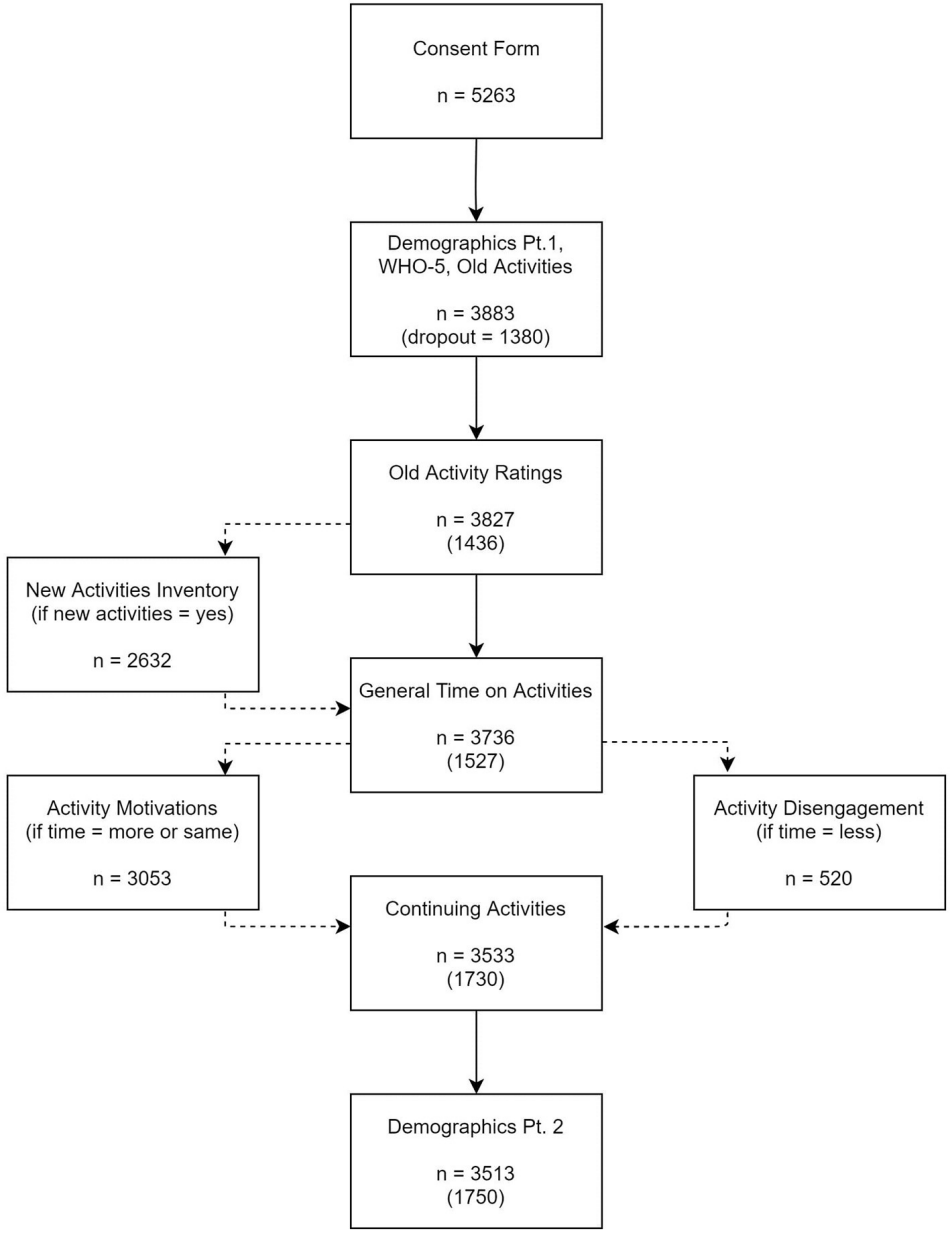
Survey sequence and attrition flowchart.

Participant attrition occurred throughout the survey. The majority of attrition occurred immediately after question eight (63.8% of total dropout), when participants were asked to list leisure activities using open-ended fields. The absence of activity data for these participants rendered their responses unworkably incomplete, and 3,827 participants who submitted data for at least one activity were retained in the final analysis (see Data Reduction and Coding). Analyses of missingness for the excluded 1,736 participants are described in the subsequent paragraph. Figure 1 depicts the survey sequence and attrition at each stage: there was further attrition throughout the survey, with the final number of participants totaling to 3,513.

Little's MCAR (“missing completely at random”) test was run for participants (*n* = 5,165) who completed the first page of demographic questions. Results indicated three patterns of missingness through the retention point, consistent with dropout occurring after each page, χ^2^(11) = 144.71, *p* < 0.001. Binomial regression indicated that demographic characteristics were significantly associated with dropout, χ^2^(13) = 176.75, *p* < 0.001: Attrition was more likely among males [comparison group: female; odds ratio (OR) = 1.85, Wald's *z* = 9.07, *p* < 0.001, 95% CI 1.62–2.12] and younger participants (OR = 0.99 per year increase above mean = 33.1 years, *z* = −4.99, *p* < 0.001, 95% CI 0.98–0.99). Results should be interpreted in light of these potential confounds.

### Materials and Measures

The survey was administered online using SurveyMonkey® (www.SurveyMonkey.com, San Mateo, California) and consisted of four sections: demographic details, the World Health Organization Five Well-Being Index, leisure activities inventory, and a leisure motivations questionnaire. Each section is described below, although not all data are reported as they do not fall within the scope of the current article. The full questionnaire can be found in the Supplementary Material.

#### Demographic Details

Demographic questions were divided between the first and final pages of the survey to minimize dropout while allowing sufficient data collection for missingness analyses (Teclaw et al., 2012; Hochheimer et al., 2016). Participants indicated their age, gender, level of educational attainment, country of residence, and social distancing status on page one. On the final page, participants provided brief household details (number of and relationship to other cohabitants and whether any household members were vulnerable to COVID-19), their employment status, and most recent occupation.

#### World Health Organization Five Well-Being Index

The World Health Organization Five Well-Being Index (WHO-5) is a short measure of global well-being (Topp et al., 2015). It consists of five items rated on a 6-point Likert scale (0–5), the sum of which is multiplied by four to yield a global score out of 100. The instrument has been used in previous psychological research to investigate well-being in relation to coping strategies, psychosocial health, and work-life balance, and has demonstrated satisfactory construct validity in a range of health-related settings (Topp et al., 2015; Yang et al., 2018). Items were presented in random order for each participant. The scale was followed by a 5-point Likert question about time perception (“compared to last year, I feel that time is passing:” 1 = *very slowly* to 5 = *very quickly*) on the same page.

#### Leisure Activities Inventory

Participants listed up to 10 pre-pandemic leisure activities (five active and five passive) that they pursued “during a typical month last year.” They were told a leisure activity was “any pastime you pursued or participated in because you wanted to,” and were provided with examples of three active (playing sports, learning languages, and woodworking) and three passive activities (watching sports, listening to music, and going to the movies). The active/passive distinction was used to classify activities during the coding stage. Response fields were open-ended and restricted to 35 characters, following the method of Friedlander and Fine (2016). On the subsequent page, leisure activities were carried forward into pre-populated fields, and participants were asked to rate whether they spent more, less, or the same amount of time on each during COVID-19 compared to the previous year. Although not as precise as requesting the exact number of hours or minutes per day on each activity, these categories were used to provide a broad-brush picture while reducing participant dropout, and parallel the categories used by Constandt et al. (2020) in their study on exercise during lockdown in Belgium.

The ratings for “old” activities were followed by the question: “Did you take up any new activities during COVID-19?” Those who answered “yes” were invited to list up to six further activities (three passive and three active) and indicate whether each was a brand new or rediscovered former interest. Finally, all participants indicated whether they felt they had generally spent more, less, or the same amount of time overall on leisure activities during COVID-19 compared to the previous year. This question resulted in two pathways: Those who answered “more” or “the same” proceeded to the leisure motivations questionnaire, and those who answered “less” were asked about barriers to leisure, as shown in Figure 1.

#### Leisure Motivations

Participants were asked to rate their agreement with 40 leisure motivations using a 5-point Likert scale (1 = *not at all* to 5 = *very much so*). Items were adapted from Crandall (1980) and Beard and Ragheb's (1983) Leisure Motivations Instrument, and included statements such as “to be creative” or “to help get the family together more.” Additional items (e.g., “to get away from the news about current events”) specific to the pandemic circumstances were also included for exploratory analysis. Item order was randomized for each participant.

#### Barriers to Leisure Engagement

Participants who reported spending less time on leisure activities during COVID-19 were asked to rate their agreement with 16 reasons on a 5-point Likert scale (1 = *does not describe me at all* to 5 = *describes me very well*). The framework for these items was adopted from Fox, Morrow-Howell, Herbers, Battista and Baum (2017) barriers to leisure in older populations (lack of opportunity, interest, companionship, and physical capability), repurposed for multiple age groups in the context of COVID-19. Item order was randomized for each participant.

#### Leisure Continuation

Leisure activity responses (both old and new/rediscovered) were carried forward for each participant into pre-populated fields. Participants were asked to rate the likelihood that they would continue each after COVID-19 on a 5-point Likert scale (1 = *definitely not* to 5 = *definitely*).

### Data Reduction and Coding

#### Data Reduction

In total, participants generated 31,389 leisure activities, reduced to 31,017 activities after removal of “null” fields (*n* = 372) such as “NA” or “nothing else” (participants were instructed to leave unused fields blank, and this was upheld for the remaining 99.6% of null responses). For the purposes of statistical analyses, activities were also excluded (*n* = 475) if participants did not indicate: (1) whether they had spent more, less, or the same amount of time on the activity during COVID-19 (for old activities); or (2) whether the activity was a brand new or rediscovered former interest (for new activities). This reduced the total activity count to 30,542. At this stage, participants who contributed only “null” activities or skipped all ratings but continued the survey (*n* = 9) were also excluded. Together with those who exited the survey prior to rating any activities (*n* = 1,427), this brought the total participant count to 3,827, as described in the section Participants.

#### Leisure Activity Coding

The remaining 30,542 leisure activities were each assigned a specific activity code, such as “houseplant gardening,” or “drawing.” A total of 243 unique activities were identified using the classification in the Leisure Activities Finder (Holmberg et al., 1997). Where no immediate match was found, data were coded using the closest match in the occupational/educational indexes of the RIASEC coding manual (Gottfredson and Holland, 1996). These activities were further grouped into 12 general domains or “activity categories” derived from the classification system used by Friedlander and Fine (2016). Of these, six categories were considered to represent “creative” activities and six representing non-creative activities. The creative categories were as follows:

- Consumption of creative content (e.g., listening to music, watching a play, and visiting an art museum)
- Fine arts (e.g., painting, drawing, and sculpting)
- Home crafts and artisanship (e.g., gardening, cooking/baking, and knitting)
- Musical and performing arts (e.g., playing an instrument, singing, and acting)
- Niche and IT interests (e.g., building miniature models, programming, and aquarium maintenance)
- Reading, writing, and languages (e.g., reading, creative writing, and translating).

The non-creative activities were as follows:

- Mind games (e.g., video games, jigsaw puzzles, and crosswords)
- Public service and leadership (e.g., club organizing, coaching, and volunteering)
- Socializing, food, and relaxation (e.g., visiting friends, watching TV/movies, and dining out)
- Sports and outdoor pursuits (e.g., running, football, and going to the gym)
- Travel (e.g., local or international travel, leisure driving)
- Unspecified work or study activity (e.g., studying, working).

A frequency count of the activities by category can be found the Supplementary Materials. The resulting 12-level categorical variable was used in subsequent statistical analyses to evaluate changes in leisure engagement during COVID-19. All activities were coded by one rater and a random sample of 1,000 activities was coded by a second rater: Interrater agreement for the 12 upper-level categories was 96.8%, and the first rater's codings were therefore accepted.

## Results

As outlined in the Introduction, the study sought to answer five key questions, each of which is addressed in a subsection below. All statistical analyses were carried out in R (4.0.2) with an alpha level of *p* < 0.05 (R Core Team, 2020).

### Descriptive Statistics

#### Participant Demographics

Participant demographics and mean well-being scores for each group are shown in Table 1. Most participants were female (65.8%), university-educated (43.8%), and living in the United States (49.8%) or the United Kingdom (25.2%). The majority of participants reported living with at least one other person (89.0%) in a household not considered clinically vulnerable to COVID-19 (55.7%). Nearly all participants reported currently or previously practicing social distancing (97.8%), with those working from home (37.4%) comprising the most represented group.

**Table 1:** Participant demographics.

		**Mean (SD)**	***N***	**WHO-5 Mean (SD)**
**Age**		33.06 (12.63)	3,827	49.34 (19.13)
		**% Sample**	***N***	**WHO-5 Mean (SD)**
**Gender**				
	Female	65.80	2,518	47.98 (19.13)
	Male	31.25	1,196	53.20 (18.59)
	Non-binary	2.12	81	36.15 (13.70)
	Undisclosed	0.73	28	46.71 (22.02)
	Other	0.10	4	37.00 (16.45)
**Education**				
	University degree	43.79	1,676	48.79 (18.41)
	Master's degree	19.05	729	50.13 (19.15)
	High school	16.67	638	50.08 (19.88)
	Vocational/professional training	10.35	396	50.94 (20.22)
	Doctorate	7.21	276	48.07 (19.87)
	Other	2.93	112	45.89 (18.80)
**Country of residence**				
	United States	49.73	1,903	49.21 (18.67)
	United Kingdom	25.24	966	48.91 (20.33)
	Canada	7.00	268	49.66 (18.59)
	Germany	2.87	110	51.31 (19.36)
	Netherlands	1.99	76	48.53 (17.63)
	Australia	1.86	71	49.01 (18.30)
	Other (68 countries)	11.31	433	50.41 (19.08)
**Practicing social distancing**				
	Yes, some restrictions lifted	64.20	2,457	49.45 (18.87)
	Yes, currently	31.67	1,212	48.98 (19.52)
	No	2.17	83	49.30 (21.34)
	Previously, all restrictions lifted	1.96	75	51.68 (18.79)
**Number of cohabitants**				
	2–4 others	46.77	1,643	48.63 (19.35)
	One other person	38.06	1,337	50.79 (19.14)
	Living alone	10.99	386	48.55 (19.11)
	5–7 others	3.91	137	47.82 (17.53)
	8–9+ others	0.29	10	49.20 (22.31)
**COVID-19 household vulnerability**				
	No	55.74	1,958	50.93 (18.99)
	Yes	38.91	1,367	47.60 (19.44)
	Unsure/undisclosed	5.35	188	46.77 (18.30)
**Employment status**				
	Employed, working from home	37.38	1,313	49.90 (18.76)
	Student	15.97	561	48.51 (18.18)
	Employed, physically attending work	14.38	505	48.62 (19.40)
	Unemployed, not receiving financial compensation	7.54	265	45.89 (19.76)
	Unemployed, receiving financial compensation	7.12	250	50.56 (17.43)
	Employed on paid leave	5.86	206	51.51 (21.06)
	Retired	4.33	152	58.71 (20.27)
	Stay-at-home parent	3.42	120	45.10 (19.95)
	Other	4.01	141	46.50 (20.24)

#### Leisure Activities

Overall, participants reported a total of 30,542 leisure activities, from which 243 unique activities were identified. These activities were further classified into the broad leisure domains specified previously (fine arts, home crafts and artisanship, etc.) for statistical analyses. The top 10 individual activities (from *n* = 243) most likely to increase and/or to be taken up as new activities during COVID-19 are shown in Table 2: the majority were creative activities. Watching TV, online streaming services, and movies comprised 20.2% of all increased activities and 7.0% of new activities. The other top increased activities included listening to music (8.4%), reading (8.3%), knitting (6.9%), and video games (6.0%). The most common new activities included sewing/embroidery (5.6%), knitting/crochet (4.8%), reading (4.5%), and baking (3.9%), although these totals describe the present sample and should not necessarily be generalized to the wider population (see Limitations). Generally, participants also wanted to continue the vast majority of activities after COVID-19, rating 72.3% of all activities a “5” or “definitely” and 87.1% of activities a “4” or “5” on the 1–5 scale.

**Table 2:** Top 10 increased time and new leisure activities during COVID-19.

**Increased old activities (*n* = 8,354)**		**New activities (*n* = 6,665)**	
**Activity**	**Total increased activities (%)**	**Activity**	**Total new activities (%)**
Watching TV/streams/movies	20.22	Watching TV/streams/movies	6.99
^*^Listening to music	8.38	^*^Sewing/embroidery	5.63
^*^Reading	8.31	^*^Knitting/crochet	4.83
^*^Knitting/crochet	6.91	^*^Reading	4.49
Video games	5.96	^*^Baking	3.90
^*^Cooking	3.67	^*^Gardening	3.74
^*^Playing instrument	3.47	^*^Languages	3.65
^*^Gardening	2.97	Running	3.54
^*^Baking	2.90	^*^Playing instrument	3.47
^*^Sewing/embroidery	2.79	Audiobook/podcast/radio	3.29

* *Indicates activities allocated to creative categories*.

### Demographic Characteristics and Leisure Engagement

The first objective was to identify demographic characteristics associated with leisure engagement during COVID-19. Participants (*n* = 3,513) who completed all demographic questions were included in the analysis. The dependent variable was a 3-level ordinal response to the item, “In general, compared to this time last year, I feel I am spending”: (1) less time; (2) the same amount of time; or (3) more time overall on leisure activities during COVID-19. Because the outcome was a categorical variable with implied order (i.e., less to more), a proportional odds model was fitted [also known as *ordinal logistic regression;* see Harrell (2015), for further discussion]. Statistical analyses were performed using the R “Ordinal” package (Christensen, 2019). Employment status, gender, age, social distancing status, and household vulnerability to COVID-19 were fitted as predictors.

Percentages are displayed in Table 3. In general, most individuals reported increasing time on leisure activities, with some variation observed. A Likelihood-ratio test of null and fitted models indicated that a model containing gender, age, social distancing status, COVID-19 household vulnerability and employment status significantly predicted leisure engagement during COVID-19, χ^2^(18) = 222.28, *p* < 0.001, AIC = 5931.80. However, the assumption of parallel lines was violated for employment status (i.e., the slopes were not consistent across all levels of the outcome). Employment status was thus refitted as a *partial proportional odds* effect, meaning that two threshold coefficients were generated comparing the likelihood of “less” to “same or more” and “less or same” to “more” (instead of one overall coefficient), improving model fit χ^2^(8) = 22.04, *p* = 0.005, AIC = 5925.73, Nagelkerke's *R*^2^ effect size = 0.08. The assumption was not violated for the other predictors. As shown in Table 4, all predictors were significant except COVID-19 household vulnerability.

**Table 3:** Demographic characteristics and leisure activity engagement.

		**More time (%)**	**Same amount of time (%)**	**Less time (%)**	***N***
**Gender**					
	Female	68.43	17.67	13.90	2,331
	Others (Non-binary, Other, Undisclosed)	64.08	25.24	10.68	103
	Male	61.72	21.96	16.31	1,079
**Social distancing status**					
	Yes, currently	69.39	17.56	13.05	1,088
	Yes, some restrictions lifted	65.79	19.65	14.56	2,280
	Previously, all restrictions lifted	64.71	13.24	22.06	68
	No	36.36	35.06	28.57	77
**Household vulnerability to COVID-19**					
	Unsure/undisclosed	69.15	17.02	13.83	188
	No	66.19	20.07	13.64	1,958
	Yes	65.91	18.14	15.95	1,367
**Employment status**					
	Unemployed, receiving financial compensation	82.80	8.80	8.40	250
	Employed on paid leave	82.52	6.31	11.17	206
	Unemployed, not receiving financial compensation	79.26	13.96	6.79	265
	Student	75.22	14.97	9.80	561
	Other	63.83	21.99	14.18	141
	Employed, working from home	61.54	22.47	15.99	1,313
	Retired	60.53	25.00	14.47	152
	Stay-at-home parent	55.83	17.50	26.67	120
	Employed, physically attending work	51.68	26.53	21.78	505

**Table 4:** Type II analysis of effects deviance table with Wald Chi-square tests.

	**Df**	**Chi-square**	***P*-value**
Gender	4	30.42	<0.001^***^
Age	1	7.35	0.007^**^
Social distancing	3	13.55	0.004^**^
COVID-19 vulnerability	2	3.03	0.215

*When fitted as a partial proportional odds effect, employment status was not computed in the analysis of effects table despite contribution to model fit; the estimated effect before refitting was χ^2^(8) = 132.99, p < 0.001 with further improvement after relaxing the assumption of parallel lines, χ^2^(8) = 22.04, p = 0.005*.

** *p < 0.05*,

*** *p < 0.001*.

Odds ratios (ORs) were computed for coefficient analyses. ORs compare the relative likelihood of an outcome to a baseline “reference” group, where OR = 1 is chance level. For example, an OR of 1.5 reflects a likelihood of 1.5 to 1, or 50% greater likelihood. In general, ORs > 1 suggest an outcome is more likely, and ORs <1 suggest an outcome is less likely. The 95% confidence interval (CI) reflects the precision of the estimate, in which a smaller range is considered more precise [see Szumilas (2010), for further discussion].

Coefficient analysis indicated that increased leisure engagement was more likely among females (reference group: male; OR = 1.50, Wald's *z* = 5.11, *p* < 0.001, 95% CI 1.28–1.75), and less likely among older individuals (OR = 0.99 for each year increase above mean = 33.2 years, *z* = −2.71, *p* = 0.007, 95% CI 0.98–1.00) and those who were not practicing social distancing (reference: currently social distancing; OR = 0.44, *z* = −3.63, *p* < 0.001, 95% CI 0.28–0.69).

For employment status, two threshold coefficients were estimated comparing the likelihood of “less time” to “same or more” and “more time” to “less or same” due to refitting as a partial proportional odds effect. Those more likely to decrease time on leisure activities included stay-at-home parents (reference: working from home; OR = 2.05, *z* = 3.24, *p* = 0.001, 95% CI 1.33–3.17) and those physically attending work (OR = 1.32, *z* = 2.05, *p* = 0.04, 95% CI 1.01–1.72). Those more likely to increase time on leisure activities (i.e., less likely to choose “same” or “less,” resulting in an OR <1) were students (reference: working from home; OR = 0.55, *z* = −4.72, *p* < 0.001, 95% CI 0.43–0.71) and those employed on paid leave (OR = 0.35, *z* = −5.40, *p* < 0.001, 95% CI 0.24–0.52), unemployed without financial compensation (OR = 0.41, *z* = −5.43, *p* < 0.001, 95% CI 0.30–0.57), and unemployed with financial compensation (OR = 0.33, *z* = −6.28, *p* < 0.001, 95% CI 0.23–0.46).

In summary, increased time on leisure activities was generally observed across all groups, but was more likely among females than males, and less likely among older persons (progressively decreasing above mean age 33.2 years) and those who were not practicing social distancing. Compared to those working from home, students, individuals employed on paid leave, and unemployed individuals were more likely to increase time on leisure activities, while those physically attending work and stay-at-home parents were more likely to decrease time on activities.

### Leisure Engagement and Activity Domains

In contrast to who was more likely to increase time on leisure activities during COVID-19, the second objective was to identify the particular leisure domains on which time was more likely to increase, looking specifically at how participation in creative activities compared to sports and outdoor pursuits. *N* = 30,542 activities were analyzed from *n* = 3,827 participants. A multilevel binomial model was fitted with “new” or “increased time” activities coded as 1 and “decreased or “same amount of time” activities coded as 0. A binomial model was fitted because “same amount of time” comprised fewer than 15% of responses and a proportional odds model violated the assumption of parallel lines. Estimates from the proportional odds model were compared with those from partial proportional odds and binomial models and approximate equivalence was observed (i.e., similar confidence intervals and no change in significance levels). The binomial model was determined to be the most parsimonious. Random intercepts were fitted for participants to account for non-independence of multiple observations (i.e., individuals usually listed more than one activity). The twelve-level factor comprising the activity categories was fitted as a predictor. The R package “lme4” was used for model building (Bates et al., 2015).

A likelihood-ratio test of null and fitted models indicated that activity category significantly predicted leisure engagement during COVID-19, χ^2^(11) = 4264.4, *p* < 0.001, with pseudo *R*^2^ effect size = 0.16 to 0.18 for the activity category effect (explaining 16–18% of the variance) and pseudo *R*^2^ = 0.21 to 0.24 (21–24% of the observed variance) for the full model containing activity category and random intercepts (*R*^2^ estimates were computed using the “MuMIn” package in *R*; Nakagawa and Schielzeth, 2012).

**Figure 2:**
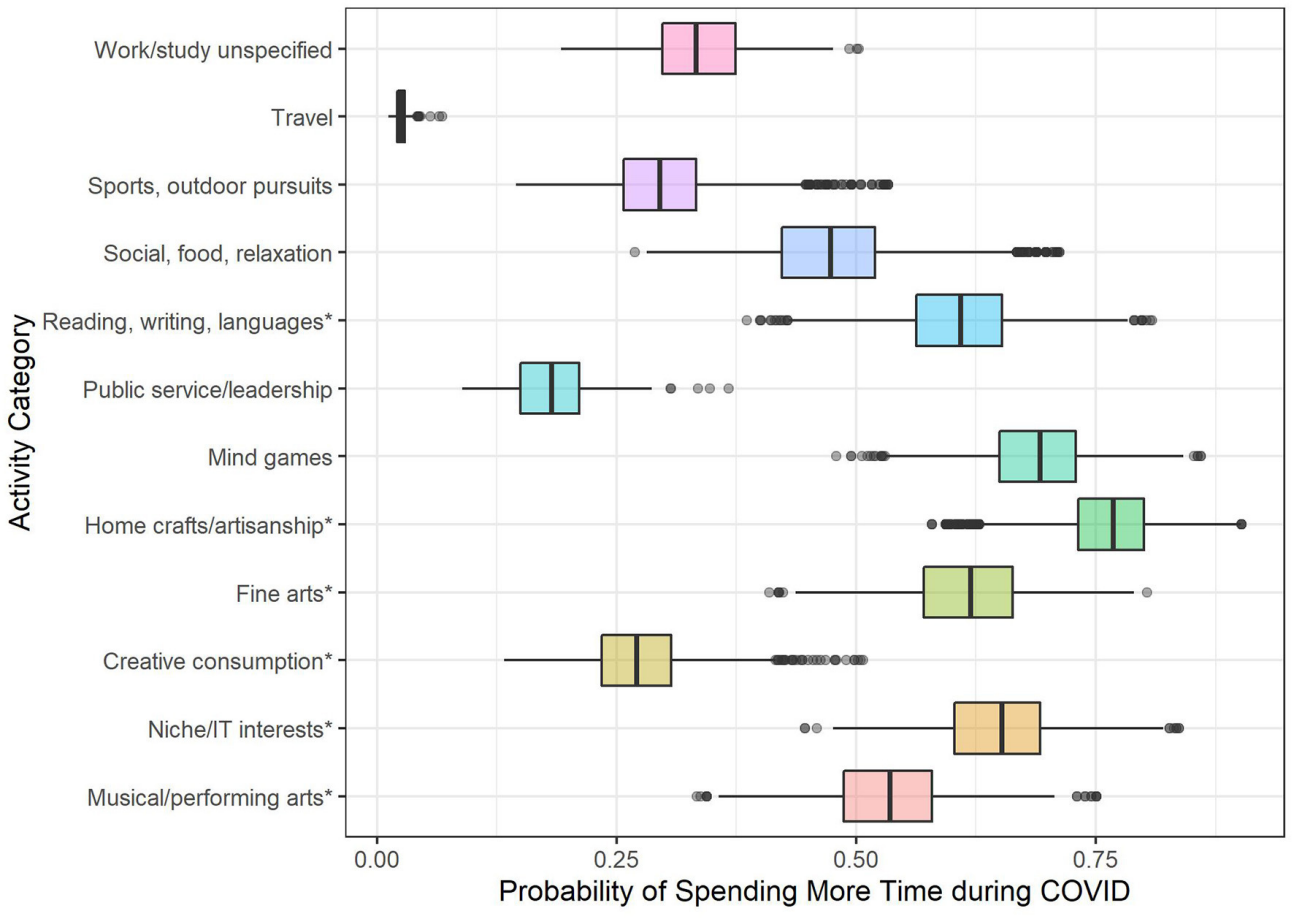
Probability of taking up or increasing time on leisure activities during COVID-19 predicted by activity category. * indicates activities allocated to creative categories.

Predicted probabilities are shown in Figure 2. “Sports and outdoor pursuits” was used as the reference level for OR estimates, as previous studies such as those reviewed above have focused primarily on engagement in physical activities. Compared to sports and outdoor pursuits, activities on which time was more likely to increase included home crafts and artisanship (OR = 8.08, Wald's *z* = 46.38, *p* < 0.001, 95% CI 7.40–8.83), mind games (OR = 5.41, *z* = 27.24, *p* < 0.001, 95% CI 4.79–6.10), niche and IT interests (OR = 4.48, *z* = 16.78, *p* < 0.001, 95% CI 3.76–5.33), fine arts (OR = 3.83, *z* = 16.67, *p* < 0.001, 95% CI 3.27–4.48), reading, writing and languages (OR = 3.69, *z* = 25.48, *p* < 0.001, 95% CI 3.34–4.08), musical and performing arts engagement (OR = 2.66, *z* = 16.01, *p* < 0.001, 95% CI 2.36–2.99), and social, food, and relaxation activities (OR = 2.16, *z* = 19.85, *p* < 0.001, 95% CI 2.01–2.34). Activities on which time was less likely to increase compared to sports and outdoor pursuits included travel (OR = 0.06, *z* = −8.88, *p* < 0.001, 95% CI 0.04–0.12) public service and leadership (OR = 0.54, *z* = −3.81, *p* = 0.001, 95% CI 0.40–0.74), and creative consumption (OR = 0.90, *z* = −2.16, *p* = 0.03, 95% CI 0.82–0.99). Engagement in unspecified work/study activities was not significantly different from sports and outdoor pursuits.

Collectively, a model comparing the likelihood of increasing creative compared with non-creative activities indicated significant differences, χ^2^(1) = 853.63, *p* < 0.001, with pseudo *R*^2^ = 0.03 for the category effect and 0.08 to 0.09 for the full model. Time on creative activities was more likely to increase than non-creative activities, OR = 2.02, Wald's *z* = 28.90, *p* < 0.001, 95% CI 1.93–2.12.

In summary, activity category was a significant predictor of leisure engagement during COVID-19. Compared to sports and outdoor physical activities, participation in creative activities including home crafts and artisanship, niche and IT interests, reading, writing and languages, fine arts, and musical and performing arts were all more likely to increase. Time spent on non-creative such as mind games and social, food, and relaxation activities were also more likely to increase. In contrast, time spent on travel, public service and leadership activities, and creative consumption were significantly less likely to increase. This suggests that participation in active creative pursuits was generally much more likely to increase during COVID-19 than participation in sports and outdoor activities, as well as more likely to increase than time on non-creative activities more generally.

### Leisure Engagement and Well-Being

The third objective was to determine whether leisure engagement (less/same/more) and participation in new leisure activities (yes/no) were associated with well-being during COVID-19 over and above potential confounding variables (age, gender, educational attainment, employment status, cohabitation, household COVID-19 vulnerability, and social distancing). These variables were selected due to their influence on subjective well-being across the lifespan (Dolan et al., 2008) or during COVID-19 (Foa et al., 2020) although their extension to the WHO-5 was not definitively known. A multiple linear regression model containing the confounds significantly predicted well-being, *F*_(24, 1)_ = 12.52, *p* < 0.001, adjusted *R*^2^ = 0.07 and the addition of time spent on activities and new activities further improved model fit, *F*_(3, 24)_ = 62.01, *p* < 0.001, adjusted *R*^2^ = 0.12, explaining an additional 5% of the variance in well-being. However, only time spent on activities significantly added to the model, while taking up new activities did not (see Table 5).

**Table 5:** WHO-5 well-being type II *F*-test analysis of effects.

	**Df**	***F* value**	***P*-value**
Age	1	90.63	<0.001^***^
Gender	4	32.51	<0.001^***^
Education	5	1.80	0.109
Employment status	8	2.92	0.003^**^
Number of cohabitants	1	3.05	0.081
COVID-19 vulnerability	2	15.94	<0.001^***^
Social distancing	3	0.57	0.634
Time on leisure activities	2	86.98	<0.001^***^
New leisure activities	1	1.41	0.235

** *p < 0.05*,

*** *p < 0.001*.

As shown in Table 5, age, gender, employment status, and COVID-19 household vulnerability were significant predictors of well-being: Coefficient estimates for these effects can be found in Supplementary Table 1. Importantly, higher levels of leisure engagement positively predicted well-being (reference group: less time; more time = 12.08 points, *t* = 13.19, *p* < 0.001, 95% CI 10.28–13.88; same amount of time = 9.77 points, *t* = 9.13, *p* < 0.001, 95% CI 7.67–11.87). In summary, increasing or maintaining levels of leisure engagement during COVID-19 predicted higher well-being.

### Leisure Domains and Well-Being

In contrast to the previous section, which established whether overall leisure engagement predicted well-being (i.e., spending more, less, or the same amount of time on activities in general), the fourth objective was to identify whether increased time on specific activities predicted well-being, taking into account the same demographic confounds. This was to determine whether engagement in creative activities predicted higher well-being outcomes, as was expected for sports and outdoor pursuits (Chtourou et al., 2020). *N* = 28,345 activities were analyzed from *n* = 3,513 participants who completed the full questionnaire. Because well-being was observed once per participant, effects were estimated at the subject level. Twelve “category scores” ranging from 0 to 1 were computed for each participant representing relative increase in leisure engagement during COVID-19.

First, individual activities were coded as 0 if participants reported spending “less” or “same amount of time” on each during COVID-19 and 1 if “more time” or “new activity,” following the coding scheme used in the “Leisure engagement and activity classification” section above. The sum of the scores for each category was then divided by the total number of activities given for that category (for example, if a participant listed 3 “arts” activities rated more [1], more [1], and less [0], their category score was 2/3 or 0.67, reflecting 67% increase in arts activities). If a participant did not provide any activities for a category, then a weight of zero was assigned. This resulted in 12 overall category scores ranging from 0 to 1, with 1 representing 100% increased time on activities in that category during COVID-19.

**Figure 3:**
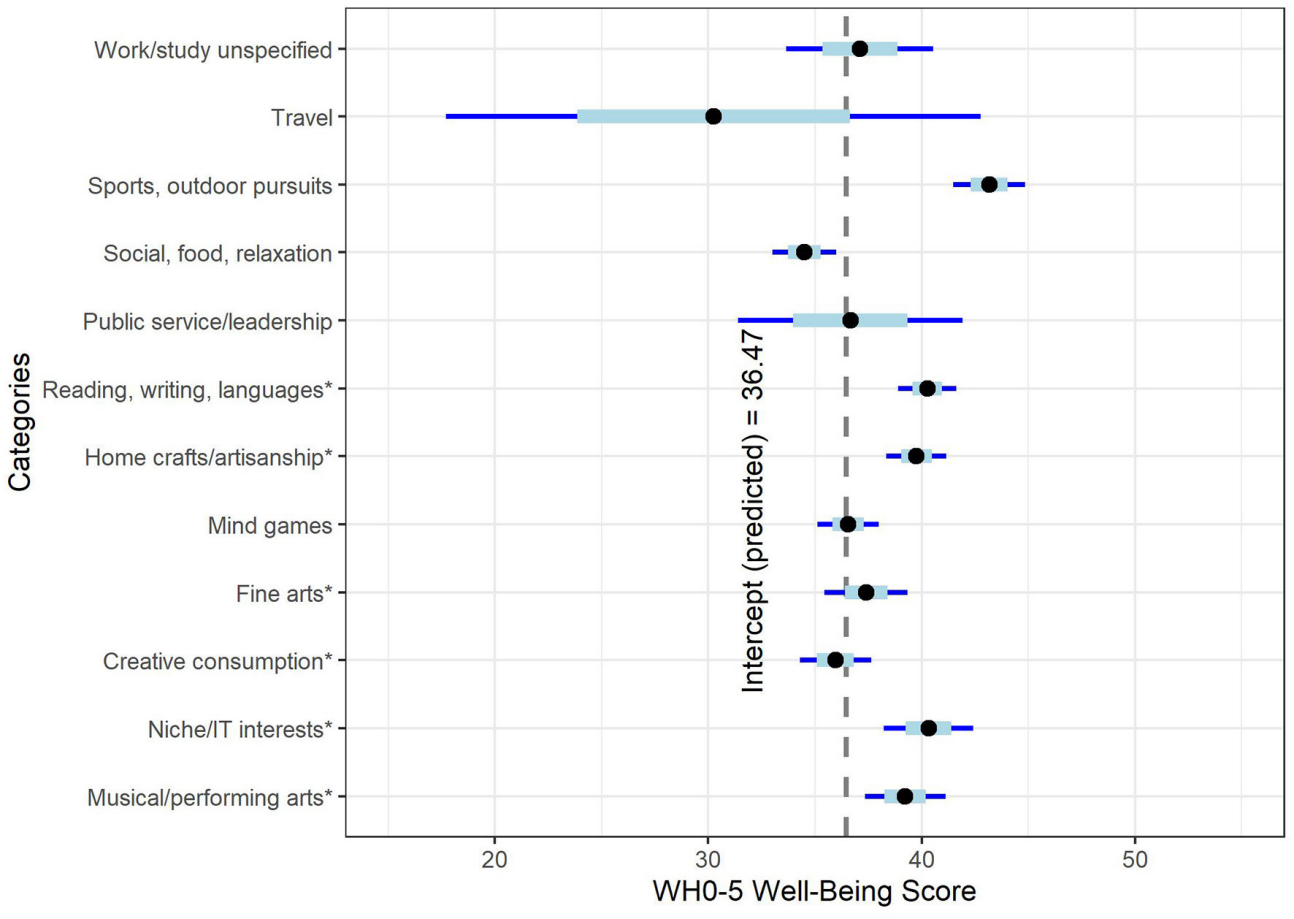
Well-being predicted by activity category. The intercept represents well-being scores when all predictors are fixed at zero. * indicates activities allocated to creative categories.

The 12 scores were fitted as predictors of well-being over and above the confound-only model using multiple linear regression, *F*_(12, 24)_ = 11.99, *p* < 0.001, adjusted *R*^2^ = 0.10. Slope coefficients are shown in Figure 3 (as very few participants reported increased time on travel activities, the large confidence interval reflects the uncertainty of this estimation). Well-being was significantly predicted by increased time on sports and outdoor pursuits (estimate = 6.69 points higher for those who increased time on all activities in this category, *t* = 7.82, *p* < 0.001, 95% CI 5.01–8.37), niche and IT interests (3.86 points, *t* = 3.61, *p* < 0.001, 95% CI 1.76–5.95), reading, writing and languages (3.79 points, *t* = 5.46, *p* < 0.001, 95% CI 2.43–5.15), home crafts and artisanship (3.29 points, *t* = 4.57, *p* < 0.001, 95% CI 1.88–4.69, and active musical and performing arts engagement (2.76 points, *t* = 2.85, *p* = 0.004, 95% CI 0.86–4.65). Increased time on social activities predicted lower well-being (−1.98 points, *t* = −2.60, *p* = 0.009, 95% CI −3.48 to −0.49), and increased time on mind games, service, work/study, fine arts, creative consumption, and traveling were not significant.

Taken collectively, creative, noncreative, and sports activities were each significant predictors of well-being, *F*_(3, 24)_ = 47.43, *p* < 0.001, adjusted *R*^2^ = 0.11. Higher well-being was predicted by increased time on creative activities (8.51 points higher, *t* = 8.51 *p* < 0.001, 95% CI 6.55–10.47) and sports and outdoor pursuits (6.70 points higher, *t* = 7.87, *p* < 0.001, 95% CI 5.04–8.38). Increased time on non-creative activities predicted lower well-being (−2.15 points lower, *t* = −2.59, *p* = 0.009, 95% CI −3.78 to −0.52).

In summary, increased time on sports and outdoor pursuits predicted the highest well-being outcomes, but increased time on creative activities such as niche and IT interests, reading, writing and languages, home crafts and artisanship, and active musical engagement also predicted significantly higher well-being.

### Leisure Motivations, Barriers to Leisure, and Well-Being

The final objective was to determine the factor structure of leisure motivations, barriers to leisure, and their association with well-being.

#### Leisure Motivations

Exploratory factor analysis using an oblique rotation was run for participants who completed the leisure motivations questionnaire (*n* = 3,053). Eight factors were identified with item loadings > 0.25 (three items, “to focus better on my work,” “to change my daily routine,” and “to think about my personal values” were removed with loadings <0.25 on all factors), explaining 40.5% of the variance in leisure motivations with good fit, TLI = 0.93, RMSR = 0.02, RMSEA = 0.03. Factors and item loadings are depicted in Table 6. Eight factors were extracted: (1) creativity and mental stimulation, which included seeking to use one's mind, practice skills and abilities, and to be creative; (2) detachment, which included physical relaxation and seeking to escape news or responsibilities; (3) competition, which included wanting to compete with or demonstrate skills to others; (4) social connection, which included maintaining contact with family/friends and helping others; (5) distraction, which included keeping busy and avoiding boredom; (6) social avoidance, which included avoiding people and the community; (7) control, which included seeking control, purpose, or to be in charge; and (8) fitness, which included exercising and being outside in natural surroundings.

**Table 6:** Leisure motivations factor analysis item loadings.

	**Creative/mental**	**Detachment**	**Competition**	**Social connection**	**Distraction**	**Social avoidance**	**Control**	**Fitness**
Practice skills and abilities	0.78							
Learn what I am capable of	0.59							
Be creative	0.56							
Be good at something	0.56							
See the results of my efforts	0.52							
Use my mind	0.51							
For the excitement	0.28							
Manage stress		0.75						
Let my mind slow down		0.73						
Improve mood		0.57						
Relax physically		0.52						
Get away from responsibilities		0.42						
Get away from news		0.33						
Make others think highly of me			0.74					
Show others I could do something			0.65					
Because of the competition			0.45					
Keep contact with family/friends				0.74				
Help bring the family together more				0.58				
Help others				0.56				
Talk to new people				0.38				
Keep busy					0.78			
Avoid boredom					0.74			
Get away from civilization						0.80		
Avoid people						0.62		
Feel as though I am in control							0.54	
Feel that I am nurturing something							0.47	
Give me a sense of purpose							0.46	
Be in charge or run things							0.40	
Exercise or keep in shape								0.74
Be close to nature								0.56

**Figure 4:**
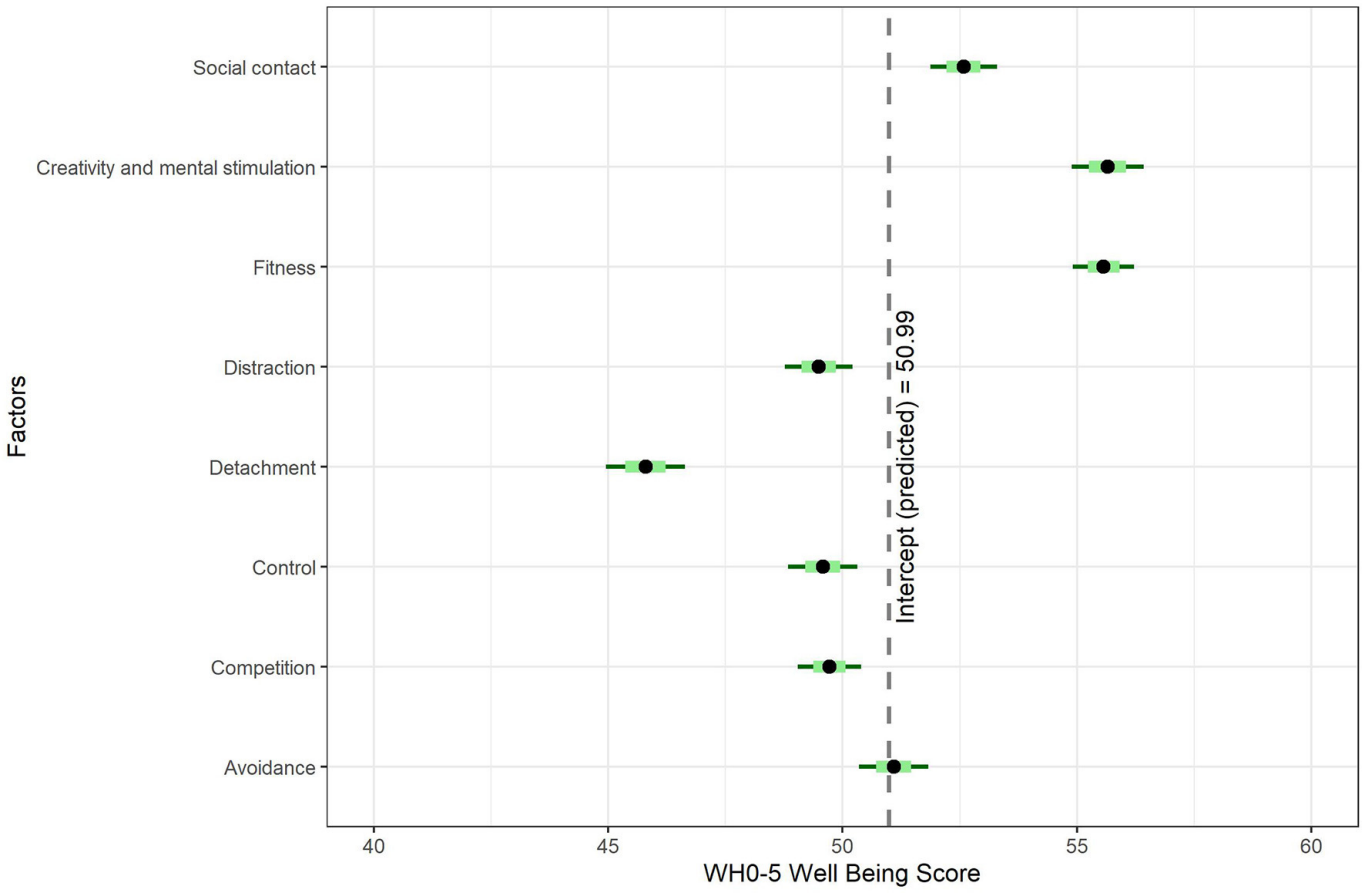
Well-being predicted by leisure motivation factor scores. The intercept represents well-being scores when all factors are fixed at zero.

Factor scores were computed using the ten Berge method for oblique rotations (ten Berge et al., 1999; Revelle, 2017) and fitted as predictors of well-being using multiple linear regression, *F*_(8, 1)_ = 83.63, *p* < 0.001, adjusted *R*^2^ = 0.18. Descriptive statistics are shown in Table 7 and coefficient estimates are shown in Figure 4. Among individuals who reported spending more or the same amount of time on leisure activities, higher scores on creativity and mental stimulation (estimate = 4.66 points higher or ~9% higher per change in standard deviation, *t* = 11.89, *p* < 0.001, 95% CI 3.89–5.43), keeping fit (4.57 points, *t* = 13.73, *p* < 0.001, 95% CI 3.92–5.23), and seeking social connections (1.59 points, *t* = 4.37, *p* < 0.001, 95% CI 0.88–2.30) predicted higher well-being. Higher scores on detachment (estimate = −5.19 points lower, *t* = −12.08, *p* < 0.001, 95% CI −6.04 to −4.35), distraction (−1.50 points, *t* = −4.08, *p* < 0.001, 95% CI −2.23 to −0.78), need for control (−1.41 points, *t* = −3.78, *p* < 0.001, 95% CI −2.15 to −0.68), and competition (−1.28 points, *t* = −3.71, *p* < 0.001, 95% CI −1.95 to −0.60) predicted lower well-being, and social avoidance was not significant.

**Table 7:** Leisure motivations factor score descriptives (*n* = 3,053).

	**Mean**	**SD**	**Median**
Distraction	4.15	1.05	4
Creativity/mental stimulation	3.88	1.17	4
Detachment	3.69	1.27	4
Control	3.10	1.44	3
Fitness	3.07	1.51	3
Avoidance	2.80	1.46	3
Social connection	2.30	1.29	2
Competition	2.04	1.27	1

*Minimum/maximum range = 1–5 for all factors*.

In summary, motivations related to seeking mental stimulation and creativity predicted the highest well-being outcomes, followed closely by keeping fit and more distantly by seeking social connections. Motivations related to feelings of mental detachment, need for control, distraction, and competition predicted lower well-being.

#### Barriers to Leisure

Exploratory factor analysis using an oblique rotation was run for those who completed the barriers to leisure questionnaire (*n* = 520). Five factors were identified with item loadings >0.25 (one item, “I have caring responsibilities [excluding childcare],” was excluded with loadings <0.25 on all factors), explaining 42.9% of the variance in barriers to leisure with good fit, TLI = 0.95, RMSR = 0.02, RMSEA = 0.04. Factors and item loadings are depicted in Table 8. Five factors were extracted: (1) mental health problems, which included feeling depressed, anxious, or having difficulty concentrating; (2) home responsibilities, which included working from home, childcare, and less free time available; (3) lack of resources, which included financial problems or inhibited access to transportation; (4) work, or physically attending one's place of work; and (5) social restrictions, which included closure of leisure facilities and limited social opportunities, particularly in relation to student life (i.e., co-occurring with studying responsibilities).

**Table 8:** Barriers to leisure factor analysis item loadings.

	**Mental health**	**Home responsibilities**	**Inadequate resources**	**Workplace duties**	**Social restrictions**
Feeling low/depressed	0.84				
Feeling less motivated	0.80				
Feeling anxious	0.70				
Difficulty concentrating	0.72				
Less free time		0.99			
Working from home		0.37		−0.32	
Childcare		0.27			−0.30
Inadequate resources			0.69		
Financial difficulties			0.67		
No transportation			0.42		
Physical health problems			0.26		
Going to work				0.79	
No one to go with					0.63
Places closed					0.33
Studying responsibilities					0.29

**Figure 5:**
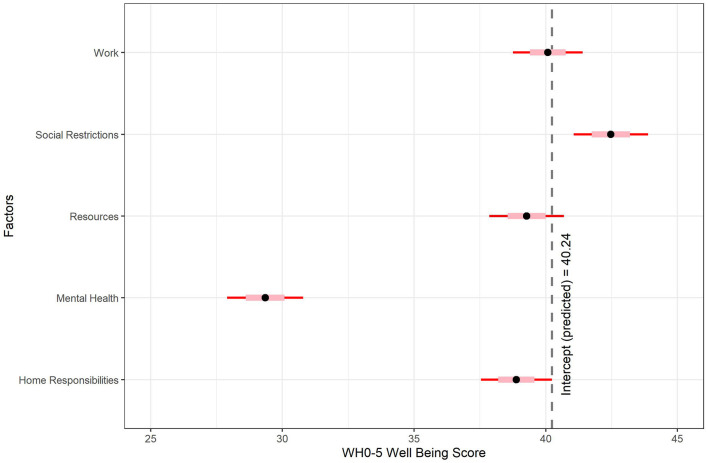
Well-being predicted by barriers to leisure factor scores. The intercept represents well-being scores when all factors are fixed at zero.

Factor scores were computed using the ten Berge estimation method for oblique rotations and fitted as predictors of well-being using multiple linear regression, *F*_(5, 1)_ = 53.40, *p* < 0.001, adjusted *R*^2^ = 0.34. Descriptive statistics are shown in Table 9 and coefficient estimates are shown in Figure 5. Among those who reported decreased time on leisure activities, mental health problems were predictive of lower well-being outcomes (−10.89 points or ~37% lower per increase in standard deviation, *t* = −14.77, *p* < 0.001, 95% CI −12.34 to −9.44), while social restrictions predicted higher well-being (2.24 points higher, *t* = 3.10, *p* = 0.002, 95% CI 0.82–3.65). The other factors were not significant. In summary, barriers related to mental health problems predicted lower well-being, while those related to social restrictions predicted higher well-being.

**Table 9:** Barriers to leisure: factor score descriptives (*n* = 520).

	**Mean**	**SD**	**Median**
Mental health	3.41	1.35	4
Home responsibilities	2.67	1.72	2
Social restrictions	2.67	1.73	2
Work	2.42	1.71	1
Resources	1.93	1.28	1

*Minimum/maximum range = 1–5 for all factors*.

## Discussion

Findings related to each of the five main research questions are discussed below, followed by a general discussion of findings, limitations, and directions for future research.

### Leisure During COVID-19: Were Demographic Variables Associated With Increased Leisure Engagement?

The first objective was to determine whether demographic variables predicted leisure engagement during COVID-19. The answer to this question was yes: While a general tendency toward spending more time on leisure activities was observed across all participants, increased engagement was particularly more likely among younger persons, females, and those currently or previously practicing social distancing. The strongest effects were observed for employment status, with >75% of students, individuals employed on paid leave, and unemployed individuals increasing time on activities, while only 50–60% of those physically attending work and stay-at-home parents reported increased time, and others (retired, working from home) falling within this range. These differences are generally consistent with previous research demonstrating that key workers (particularly those of lower income brackets) were less likely to experience additional free time during COVID-19 (Office of National Statistics, 2020), as well as lower levels of physical activity during COVID-19 reported among males (Maugeri et al., 2020; Smith et al., 2020) and older individuals (Constandt et al., 2020). The present study extended these findings to leisure time more broadly (i.e., including not only fitness but also creative pursuits) and identified stay-at-home parents as an additional group facing potential obstacles to leisure during the COVID-19 pandemic.

However, it should be noted that the overall effect of the demographic predictors was modest, explaining 8% of the variance in leisure engagement, and >50% of participants in all groups reported increased time on activities (excluding those not practicing social distancing at 36.4%). On one hand, it may be that the survey was completed by those with additional free time for leisure. However, this finding may also reflect a tendency toward increased time on leisure activities as a result of COVID-19, likely influenced by increased working from home, reduced commuting hours, and other stay-at-home mandates (Office of National Statistics, 2020; Sen-Crowe et al., 2020).

### Leisure Engagement During COVID-19: Were Certain Activities More Likely to Increase?

The second objective aimed to determine whether participation in particular types of leisure activities was more likely to increase during COVID-19, looking specifically at how creative activities compared to sports and outdoor pursuits (i.e., physical activities), as changes in the pursuit of physical activity have received extensive coverage in prior research. Findings revealed that creative activities including home crafts and artisanship, niche and IT interests, language activities, fine arts, and musical and performing arts were all more likely to increase or to be taken up as new activities during COVID-19 than sports and outdoor pursuits. Non-creative pursuits including mind games and social, food and relaxation were also more likely to increase than physical activities, whilst time spent on creative consumption, travel, work/study, and public service activities were less likely to increase. Moreover, when time on creative activities (as a composite) was compared with non-creative activities, individuals were more than twice as likely to increase time on creative than non-creative pursuits.

Broadly, these results support previous findings that time on DIY and gardening, social media, games, entertainment, and reading increased during COVID-19 (Balhara et al., 2020; Lades et al., 2020; Office of National Statistics, 2020; Rodriguez-Rey et al., 2020), but extend these findings to creative pursuits such as arts and musical engagement. Moreover, specific activities such as knitting, sewing, baking, and playing musical instruments were among those most likely to be taken up or increased during COVID-19, although this may be confounded by the total volume of these activities (see Limitations). These findings suggest that, despite widespread media coverage on engagement in athletic and outdoor activities during the COVID-19 pandemic, participation in creative pursuits was in fact more likely to increase during this period (Bryant, 2020; Minsberg, 2020). Of course, these changes are not entirely a reflection of personal agency: The closure of gyms, leisure centers, and other athletic facilities in many countries directly impacted the ability to pursue these activities. Similarly, opportunities to engage in “creative consumption” activities such as attending theater or watching a concert were in many cases no longer possible. However, both classes of activities had comparable alternatives, e.g., home workouts or running, attending online concerts, etc., and increased engagement was still more prominent for creative pursuits. On the other hand, travel and public service activities were less likely to increase, almost certainly because these activities were the most affected by travel restrictions, social distancing requirements, and/or the closure of leisure facilities (Gossling et al., 2020).

For sports and outdoor pursuits, previous research on lockdown in Belgium reported that physical activities decreased among those who were highly active but increased among those who were previously inactive (Constandt et al., 2020). Results of the present study found that physical activities were generally less likely to increase, but did not consider pre-pandemic levels of physical activity. These findings are consistent with a US study reporting that only 27% of individuals increased time on physical activities during March and April 2020 (Duncan et al., 2020), as well as concerns raised regarding increased sedentary behavior during COVID-19 (Narici et al., 2020; Pecanha et al., 2020). However, an additional reason that fitness activities may have been less likely to increase was due to the consolidation of all activities (*n* = 243) into 12 categories. Sports and outdoor pursuits included, for example, activities such as going to the gym (generally more likely to decrease due to the closure of public facilities in many countries/regions) and running (more likely to increase, as noted above), resulting in reduced likelihood overall. Thus, while single activities such as running were identified among those most likely to be pursued during COVID-19, these were not necessarily consistent with the overall trends observed in each category. More detailed investigation of subcategories may reveal differences at a higher level of specificity, but the aim of the present analysis was to characterize general trends. Notably, these trends also represented patterns over a period of several months (i.e., March/April through early June 2020) compared to previous studies which typically investigated behavior during the early stages of COVID-19 in March and April 2020 (e.g., Lades et al., 2020).

Broadly speaking, the activities most likely to be taken up or increased during COVID-19 were those that could be easily pursued from home, consistent with research showing increased monetary spending on activities such as DIY and gardening during COVID-19 (Leatherby and Gelles, 2020) and studies on leisure pursuits during lockdown (Rodriguez-Rey et al., 2020), with the present study extending these findings to other creative activities including arts and language pursuits. Moreover, while previous studies typically looked at the immediate impact of lockdown over a 24-h period (e.g., in countries such as Ireland; Lades et al., 2020), participants in the present study were asked to recall typical activities pursued during the previous year, to assess their current engagement in these activities (from March/April through early June 2020), and to identify any new leisure pursuits in recent months. The present study thus established not only how people were spending time during COVID-19, but specifically how engagement in these activities compared to life before the pandemic, with creative activities such home crafts and artisanship, niche and IT interests, language pursuits, creative arts, and musical engagement all more likely to be taken up or increased than sports and outdoor pursuits and non-creative activities. These changes reflect a combination of both the direct impact of COVID-19 on access to various activities (e.g., closure of leisure facilities, limitations on social interaction, etc.) as well as personal decisions to undertake various activities where similar restrictions did not apply.

### Leisure and Well-Being: Did Increased Time on Leisure Activities Predict Well-Being Over and Above Demographic Confounds?

The third and fourth objectives were to determine whether increased time on leisure (in general), the uptake of new leisure activities, and increased time on specific activities predicted well-being over and above potential confounding variables such as age and employment status. Results indicated that while taking up new activities was not predictive of well-being, higher well-being was predicted for those who maintained or increased time on activities during COVID-19, compared to those who decreased time. However, implications of causality remain unclear: While some perspectives posit that engagement in leisure activities may offer specific benefits for well-being (Newman et al., 2014; Kuykendall et al., 2018), it is equally plausible that these differences suggest that those with higher well-being had more time or motivation to pursue leisure activities. Most likely, it is a combination of both, with leisure engagement and well-being reciprocally affecting one another. That is to say, no definitive conclusions can be drawn regarding the potential impact of leisure activities on well-being (or vice versa) based on the methods of the present study.

As overall time on leisure activities predicted well-being during COVID-19, the fourth objective was to determine whether increased time on specific activities was associated with higher well-being. Results suggested that increased time on sports and outdoor pursuits, home crafts and artisanship, language activities, niche and IT interests, fine arts, and musical and performing arts engagement each predicted higher well-being. Predictions ranged from ~8% higher for musical engagement to 21% for sports and outdoor pursuits, with other activities falling within this range. On the other hand, increased time on social, food, and relaxation activities (e.g., activities such as watching movies or dining out) predicted lower well-being. Creative activities (taken collectively) and engagement in sports and outdoor pursuits were each linked to higher well-being outcomes, whereas increased time on non-creative activities was linked to lower well-being. These results are consistent with previous findings showing psychological benefits associated with fitness and gardening activities during COVID-19 (Lades et al., 2020), but extend to other pursuits such as creative arts, musical engagement, niche and IT interests, and language activities, and suggests that these benefits hold for both physical and creative activities, but not non-creative activities. While not specifically in the context of COVID-19, prior research has highlighted the benefits of musical engagement (Fritz and Avsec, 2007; Hallam et al., 2014), reading (Billington et al., 2010), and other visual and movement-based creative arts activities (Stuckey and Nobel, 2010) on mental health, consistent with these findings. The present study provides additional support for the positive relationship between engagement in creative activities and mental health, and suggests that these benefits may also extend to the circumstances of the COVID-19 pandemic.

While results should not be taken to suggest that increasing time on these activities necessarily influenced well-being, these findings indicate that higher well-being was observed among those who increased time on these activities. Additional factors such as home ownership and access to outdoor space may, for example, affect both well-being and the ability to pursue some activities such as gardening and outdoor sports (Barton and Rogerson, 2017; Munford et al., 2020), but not necessarily others such as language activities or creative arts. Importantly, an abundance of research has highlighted the negative impacts of COVID-19 on mental health (Qiu et al., 2020; Rajkumar, 2020), as well as the psychological benefits of engaging in physical activities (Carriedo et al., 2020; Chtourou et al., 2020). The present findings suggest that creative pursuits may help to facilitate the promotion of psychological well-being during COVID-19, though causal direction remains uncertain. Moreover, these findings warrant further investigation of the potential psychological benefits of engaging in creative leisure in non-pandemic times.

### Leisure Motivations and Well-Being: Were Leisure Motivations and Barriers to Leisure Predictive of Well-Being?

The fifth objective was to identify the overarching motivations for and barriers to leisure engagement during COVID-19 and their relationship to psychological well-being. As demonstrated above, most participants reported increased participation in leisure activities during COVID-19, but the reasons for this ranged from occupying additional free time to seeking new creative outlets. Overall, eight primary motivations were identified, higher scores on three of which predicted higher well-being (creativity and mental stimulation, keeping fit, and social connections) and higher scores on four of which predicted lower well-being (detachment, distraction, competition, and need for control) and one was not significant (social avoidance).

These motivations exhibited considerable overlap with the psychological mediators of leisure and well-being posited in the DRAMMA model (work detachment-recovery, autonomous motivation, mastery and skill development, meaning-making, and social affiliation) and suggested that comparable mechanisms may be underpinning the relationship between leisure engagement and well-being during COVID-19 (Newman et al., 2014). However, it is notable that not all motivations identified had a positive impact on well-being. For example, previous research has suggested that increasing time on activities such as video games and fitness pursuits as a coping mechanism for anxiety may not be enough to reduce stress associated with the pandemic (Balhara et al., 2020; Duncan et al., 2020). Indeed, in the present study, mental detachment (i.e., avoidance of unpleasant thoughts and stress reduction) motivations were associated with lower well-being, predicting a 10% decrease in well-being as scores on this factor increased. On the other hand, reasons such as seeking creativity and mental stimulation and keeping fit were associated with the highest outcomes, each predicting a 9% increase in well-being as scores on these factors increased. Thus, these findings suggest that not only the types of activities pursued during COVID-19 predicted higher well-being, but also why such activities were undertaken.

Importantly, motivations related to keeping fit and seeking creativity and mental stimulation predicted an equivalent increase in well-being, suggesting benefits of both for psychological health during COVID-19. While the categorization of activities into “creative” and “non-creative” domains was determined based on external criteria, this finding suggests that specifically the subjective *experience* of being creative was equally as beneficial for well-being as motivation to keep fit, highlighting the impact of creative motivations on mental health during the COVID-19 pandemic. Previous literature has shown that the primary motivation for engaging in everyday creative activities is enjoyment, with specific activities such as cooking and handicrafts reflecting prosocial motives and visual and musical arts reflecting the need for self-expression (Benedek et al., 2019). Although the present study did not look at motivations in relation to specific activities, this may suggest that creative activities likewise helped to fulfill these needs during the COVID-19 pandemic. However, data were only collected from those who reported maintaining or increasing time on activities (*n* = 3,053) during COVID-19, and estimates may have been further improved by extending the analysis to all participants.

Similarly, analysis of barriers to leisure among those who decreased time on activities revealed five primary obstacles: attending work, lack of resources, home responsibilities (including working from home), mental health problems, and restrictions on social activity. Mental health problems predicted significantly lower well-being (~27% lower as scores on this barrier increased), while restriction on social activity predicted higher well-being (~5% higher as scores on this barrier increased). As before, data were only collected from those who reported decreased time on leisure activities, and extending the analysis to all participants may have highlighted further differences between those who increased, decreased, or spent the same amount of time on leisure during COVID-19. Importantly, these findings demonstrate that mental health problems were a significant predictor of lower well-being during COVID-19, consistent with previous findings (Banks and Xu, 2020; Qiu et al., 2020), and suggest that mental health problems may also act as a barrier to leisure engagement during COVID-19.

### General Discussion and Implications

The COVID-19 pandemic has been associated with a rise in mental health problems including depression, anxiety, and increased stress levels (Rajkumar, 2020; Torales et al., 2020). At the same time, widespread transition to remote employment throughout 2020 saw ~40–50% of US and UK employees working at least partially from home, leaving many with additional free time that would ordinarily be spent commuting or working (Office of National Statistics, 2020; Wong, 2020). Similarly, others may have found themselves with additional free time due to employment layoffs, mandatory paid leave, and/or the closure of schools and other facilities (Golberstein et al., 2020; Lemieux et al., 2020). In the midst of pandemic-related stress and uncertainty, leisure pursuits may act as a refuge for mental health, allowing individuals to become immersed in personally meaningful activities, or by helping to promote work-life balance in a world where work and home are often indistinguishable (Buettner et al., 2011; Hayes et al., 2020). To date, an abundance of research has highlighted the psychological benefits of engaging in physical leisure activities during COVID-19 (Carriedo et al., 2020; Chtourou et al., 2020), but much less attention has been given to the potential benefits of creative pursuits.

In the present study, participants generally reported spending more time on leisure activities during COVID-19 (with some variation observed based on gender, age, social distancing practices, and employment status), similar to previous studies, with participation in activities such as home crafts and artisanship, mind games, languages, social/food and relaxation, niche and IT interests, fine arts, and music and performing arts all more likely to increase than sports and outdoor pursuits (Lades et al., 2020; Rodriguez-Rey et al., 2020). Taken together, creative activities were also more likely to be taken up than non-creative activities. Importantly, increased time on activities predicted well-being over and above demographic variables, with participation in sports and outdoor pursuits, home crafts and artisanship, language activities, niche and IT interests, fine arts, and musical and performing arts engagement each predicting higher well-being outcomes. Although fitness pursuits and creative activities were both associated with higher well-being, increased time on non-creative activities was not. Finally, motivations for pursuing leisure such as seeking creativity and mental stimulation, keeping fit, and pursuing social connections also predicted higher well-being, while mental health problems and seeking distraction and detachment predicted lower well-being. This suggests that, regardless of the specific activities undertaken, the motivation to “be creative” in one's leisure pursuits was equally as beneficial for well-being as motivations to keep fit during the COVID-19 pandemic, and may warrant further investigation into the impact of creative motivations for undertaking leisure activities independently of the pandemic.

Taken together, these findings suggest that engaging in leisure activities during COVID-19 has likely had protective benefits for psychological health, and that these benefits extend to both physical activities and creative pursuits. While participating in physical activity is crucial for optimal health and well-being (Narici et al., 2020), creative activities may also provide opportunities to nourish the mind during COVID-19 home isolation. However, causal inferences remain uncertain. For some, it may be that leisure engagement has served as a coping mechanism for stress associated with “working from home and living at work” in the unique context of COVID-19 (Hayes et al., 2020, p. 5). For others, the abrupt changes in work structure may have helped to promote a healthier work-life balance by allowing additional time for meaningful leisure pursuits. As participants reported wanting to continue over 85% of all activities in post-pandemic life, it remains to be seen whether continued engagement in these activities will offer extended benefits for mental health, or whether activities will be discontinued as time and motivation dwindle in the return to normality. Of importance here is whether, post-pandemic, creative activities will be recognized to be as beneficial and worthwhile for overall health and well-being as physical activity is generally advised to be (Sport England, 2020; World Health Organization, 2020b). If so, this would imply that at a governmental and international level financial and societal support for the pursuit of creative activities should be prioritized alongside support for physical activities. If this is not the case, then some of those who plan on continuing creative pursuits post-lockdown may not have the opportunity to do so. In either case, the present study suggests that leisure engagement has likely offered protective benefits for well-being during COVID-19, supporting prior evidence on the psychological benefits of physical activity during COVID-19 and extending these findings to creative pursuits. With the potential for further outbreaks in the future, these results suggest that strategies to encourage participation in creative leisure activities should also be considered in future guidance on the promotion of mental health during periods of lockdown or isolation, and potentially into life beyond the pandemic.

### Limitations and Future Research

Several limitations of the present research should be noted. As the survey was administered online, response rates were likely affected by self-selection biases, that is, completed by those interested in leisure during COVID-19 or who had additional time to do so (Bethlehem, 2010). Moreover, because the survey was circulated and reshared through online platforms, the prevalence of certain activities may be greater or less than that observed in the general population (i.e., reshared among those with common interests). Similarly, the majority of respondents reported residing in North America or Europe, especially the United States and United Kingdom, and findings may not necessarily generalize to the global population. Thus, more research is needed on leisure during COVID-19 in regions outside of these areas.

Limitations of the measurement instruments should also be considered. First, the coding scheme for the leisure activity categories and the distinction between creative/non-creative was derived from the RIASEC coding manual (Gottfredson and Holland, 1996) and previous literature on creative theory (Kaufman and Beghetto, 2009), but both the categories themselves and the application of the coding scheme involve subjective determinations of what is creative, active, etc. For several questions, multiple-choice options were used to reduce participant dropout at the expense of detail. For example, Likert-scale categories (less-same-more) were used to evaluate leisure engagement. These encompassed a wide range of possible values, with “more” potentially representing, for example, an increase of either 30 min or 3 h daily. Measuring time in estimated minutes/hours per day or week may have provided more informative insights into the range of differences between individuals, although the same overall trends would have been anticipated. However, because the majority of participant dropout (>60%) occurred during the open-ended activity fields immediately preceding this section, Likert-scale categories were used to mitigate further dropout while maximizing data collection for statistical analyses. For similar reasons, the WHO-5 was used to measure well-being, which may not be as extensive as other multidimensional measures of subjective well-being [see Cummins et al. (2009), for a review]. Finally, participants completed either the leisure motivations questionnaire (if spending same/more time overall on activities) or the barriers to leisure questionnaire (if spending less time) but not both. Requesting all participants to complete both questionnaires would have further improved analyses (at the expense of increased dropout), allowing further investigation into the relationship between leisure motivations and barriers directly.

Consideration of these limitations, as well as further investigation of leisure subcategories (e.g., playing music in a band vs. practicing an instrument at home) may help to provide a more comprehensive picture of the relationship between leisure and well-being during COVID-19. Additionally, it remains to be seen whether participants' intent to continue activities will manifest over time, and whether further differences will emerge between the pursuit of physical and creative activities. Perhaps most important are the remaining questions about the impact of creative motivation: Did creative leisure activities simply help to fulfill psychological needs left unmet by the pandemic, or did engagement in creative activities and specifically the motivation of “being” creative enhance well-being in a meaningful, enduring manner? Should guidance on maintaining well-being encourage the pursuit of both physical activities to nourish the body and creative pursuits for the mind? Future research investigating the impact of leisure engagement after COVID-19 will be needed to determine whether continuing these activities offers extended benefits for psychological well-being in post-pandemic life.

## Data Availability Statement

The raw data supporting the conclusions of this article will be made available by the authors, without undue reservation.

## Ethics Statement

The studies involving human participants were reviewed and approved by School of Psychology and Wellbeing Ethics Committee, University of Buckingham. The patients/participants provided their written informed consent to participate in this study.

## Author Contributions

KM, KF, and PF contributed to the study conceptualization and design. KM coded the leisure activity data, performed the statistical analyses, and wrote the paper. KF conducted inter-rater coding. KF and PF provided critical feedback to shape the final manuscript. All authors discussed their interpretation.

## Conflict of Interest

The authors declare that the research was conducted in the absence of any commercial or financial relationships that could be construed as a potential conflict of interest.

## References

[B1] Adams K. Leibbrandt S. Moon H. (2011). A critical review of the literature on social and leisure activity and well-being in later life. Aging Soc. 31, 683–712. 10.1017/S0144686X10001091

[B2] Balhara Y. Kattula D. Singh S. Chukkali S. Bhargava R. (2020). Impact of lockdown following COVID-19 on the gaming behavior of college students. Ind. J. Publ. Health 64, S172–S176. 10.4103/ijph.IJPH_465_20 32496250

[B3] Banks J. Xu X. (2020). The mental health effects of the first two months of lockdown and social distancing during the Covid-19 pandemic in the UK. Instit. Fiscal Stud. 2020:12239. 10.1111/1475-5890.12239

[B4] Barton J. Rogerson M. (2017). The importance of greenspace for mental health. Br. J. Psychiatry Int. 14, 79–81. 10.1192/S2056474000002051 PMC5663018 29093955

[B5] Bates D. Machler M. Bolker B. Walker S. (2015). Fitting linear mixed-effects models using lme4. J. Statist. Softw. 67, 1–48. 10.18637/jss.v067.i01

[B6] Beard J. G. Ragheb M. G. (1983). Measuring leisure motivation. J. Leisure Res. 15, 219–228. 10.1080/00222216.1983.11969557

[B7] Benedek M. Bruckdorfer R. Jauk E. (2019). Motives for creativity: exploring the what and why of everyday creativity. J. Creative Behav. 54, 610–625. 10.1002/jocb.396

[B8] Bethlehem J. (2010). Selection bias in web surveys. Int. Statist. Rev. 78, 161–188. 10.1111/j.1751-5823.2010.00112.x

[B9] Billington J. Dowrick C. Hamer A. Robinson J. Williams C. (2010). An Investigation Into the Therapeutic Benefits of Reading in Relation to Depression and Well-Being. Liverpool Health Inequalities Research Institute. Available online at: https://www.rm.dk/api/NewESDHBlock/DownloadFile?agendaPath=%5C%5CRMAPPS0221.onerm.dk%5CCMS01-EXT%5CESDH%20Data%5CRM_Internet%5CDagsordener%5CUdvalg_vedroerende_ku%202015%5C01-10-2015%5CAaben_dagsorden&appendixId=116564 (accessed June 08, 2021).

[B10] Braus M. Morton B. (2020). Art therapy in the time of COVID-19. Psychol. Trauma Theory Res. Pract. Pol. 12, S267–S268. 10.1037/tra0000746 32478544

[B11] Brooks S. Webster R. Smith L. Woodland L. Wessely S. Greenberg N. Rubin G. (2020). The psychological impact of quarantine and how to reduce it: rapid review of the evidence. Lancet 395, 918–920. 10.1016/S0140-6736(20)30460-8 32112714 PMC7158942

[B12] Bryant M. (2020). Cycling “Explosion:” Coronavirus Fuels Surge in US Bike Ridership. The Guardian. Available online at: https://www.theguardian.com/us-news/2020/may/13/coronavirus-cycling-bikes-american-boom (accessed June 08, 2021).

[B13] Buecker S. Simacek T. Ingwersen B. Terwiel S. Simonsmeier B. (2020). Physical activity and subjective well-being in healthy individuals: a meta-analytic review. J. Health Psychol. Rev. 2020:1760728. 10.1080/17437199.2020.1760728 32452716

[B14] Buettner L. Shattell M. Reber M. (2011). Working hard to relax: improving engagement in leisure time activities for a healthier work-life balance. Issues Mental Health Nurs. 32, 269–270. 10.3109/01612840.2011.553346 21355763

[B15] Carr S. (2020). Art therapy and COVID-19: supporting ourselves to support others. Int. J. Art Therapy 25, 49–51. 10.1080/17454832.2020.1768752

[B16] Carriedo A. Cecchini J. Fernandez-Rio J. Mendez-Gimenez A. (2020). COVID-19, psychological well-being and physical activity levels in older adults during the nationwide lockdown in Spain. Am. J. Geriatric Psychiatry. 8:7. 10.1016/j.jagp.2020.08.007 32919872 PMC7443087

[B17] Chinazzi M Davis J. Ajelli M. Gioannini C. Litvinova M. Merler S.. (2020). The effect of travel restrictions on the spread of the 2019 novel coronavirus (COVID-19) outbreak. Science 368, 395–400. 10.1126/science.aba9757 32144116 PMC7164386

[B18] Christensen R. (2019). Cumulative Link Models for Ordinal Regression With the R Package Ordinal. Comprehensive R Archive Network. Available online at: https://cran.r-project.org/web/packages/ordinal/vignettes/clm_article.pdf (accessed June 08, 2021).

[B19] Chtourou H. Trabelsi K. H'mida C. Boukhris O. Glenn J. Brach M.. (2020). Staying physically active during the quarantine and self-isolation period for controlling and mitigating the COVID-19 pandemic: a systematic overview of the literature. Front. Psychol. 11:1708. 10.3389/fpsyg.2020.01708 33013497 PMC7466737

[B20] Clark C. Picton I. Lant F. (2020). “More Time on My Hands”: Children and Young People's Writing During the COVID-19 Lockdown in 2020. National Literacy Trust. Available online at: https://cdn.literacytrust.org.uk/media/documents/Writing_during_the_COVID-19_lockdown_report.pdf (accessed June 08, 2021).

[B21] Conner T. DeYoung C. Silvia P. (2016). Everyday creative activity as a path to flourishing. J. Posit. Psychol. 12, 181–189. 10.1080/17439760.2016.1257049

[B22] Constandt B. Thibaut E. de Bosscher V. Scheerder J. Ricour M. Willem A. (2020). Exercising in times of lockdown: an analysis of the impact of COVID-19 on levels and patterns of exercise among adults in Belgium. Int. J. Environ. Res. Publ. Health 17:4144. 10.3390/ijerph17114144 32532013 PMC7312512

[B23] Courtemanche C. Garuccio J. Le A. Pinkston J. Yelowitz A. (2020). Strong social distancing measures in the United States reduced the COVID-19 growth rate. Health Affairs 39, 1237–1246. 10.1377/hlthaff.2020.00608 32407171

[B24] Crandall R. (1980). Motivations for leisure. J. Leisure Res. 12, 45–54. 10.1080/00222216.1980.11969418

[B25] Cummins R. Mellor D. Stokes M. Lau A. (2009). “Measures of subjective well-being,” in Rehabilitation and Health Assessment: Applying ICF Guidelines, eds Mpofu E. Oakland T. (Springer), 409–423. Available online at: http://dro.deakin.edu.au/view/DU:30029082 (accessed June 08, 2021).

[B26] Dolan P. Peasgood T. White M. (2008). Do we really know what makes us happy? A review of the economic literature on the factors associated with subjective well-being. J. Econ. Psychol. 29, 94–122. 10.1016/j.joep.2007.09.001

[B27] Duncan G. Avery A. Seto E. Tsang S. (2020). Perceived change in physical activity levels and mental health during COVID-19: findings among adult twin pairs. PLoS ONE 15:e0237695. 10.1371/journal.pone.0237695 32790745 PMC7425865

[B28] Foa R. Gilbert S. Fabian M. (2020). COVID-19 and Subjective Well-Being: Separating the Effects of Lockdowns From the Pandemic. Bennett Institute for Public Policy. Available online at: https://www.bennettinstitute.cam.ac.uk/publications/covid-19-and-subjective-well-being/ (accessed June 08, 2021).

[B29] Ford D. Storey M.-A. Zimmermann T. Bird C. Jaffe S. Maddila C. (2020). A tale of two cities: software developers working from home during the COVID-19 pandemic. ArXiv. https://arxiv.org/pdf/2008.11147.pdf

[B30] Fox K. Morrow-Howell N. Herbers S. Battista P. Baum C. (2017). Activity disengagement: understanding challenges and opportunities for reengagement. Occup. Therapy Int. 2017:1983414. 10.1155/2017/1983414 29097960 PMC5612609

[B31] Friedlander F. Fine P. (2016). The grounded expertise components approach in the novel area of cryptic crossword solving. Front. Psychol. 7:567. 10.3389/fpsyg.2016.00567 27199805 PMC4853387

[B32] Fritz B. Avsec A. (2007). The experience of flow and subjective well-being of music students. Horizons Psychol. 16, 5–17.

[B33] Giordano F. Scarlata E. Baroni M. Gentile E. Puntillo F. Brienza N.. (2020). Receptive music therapy to reduce stress and improve wellbeing in Italian clinical staff involved in COVID-19 pandemic: a preliminary study. Arts Psychotherapy 70:101688. 10.1016/j.aip.2020.101688 32834302 PMC7361107

[B34] Golberstein E. Wen H. Miller B. (2020). Coronavirus disease 2019 (COVID-19) and mental health for children and adolescents. J. Am. Med. Assoc. Pediatr. 2020:1456. 10.1001/jamapediatrics.2020.1456 32286618

[B35] Goldman D. (2020). Initial observations of psychological and behavioral effects of COVID-19 in the United States, using Google trends data. SocArXiv [preprint]. 10.31235/osf.io/jecqp

[B36] Gossling S. Scott D. Hall C. (2020). Pandemics, tourism and global change: a rapid assessment of COVID-19. J. Sustain. Tour. 2020:1758708. 10.1080/09669582.2020.1758708

[B37] Gottfredson G. Holland J. (1996). Dictionary of Holland Occupational Codes, 3rd Edn. Odessa, FL: Psychological Assessment Resources.

[B38] Hallam S. Creech A. Varvarigou M. McQueen H. Gaunt H. (2014). Does active engagement in community music support the well-being of older people? Arts Health 6, 101–116. 10.1080/17533015.2013.80936923308006

[B39] Harrell F. (2015). Ordinal logistic regression. In Regression modeling strategies. Springer Ser. Statist. 7:13. 10.1007/978-3-319-19425-7_13

[B40] Hayes S. Priestley J. Ishmakhametov N. Ray H. (2020). “I'm not working from home, I'm living at work:” perceived stress and work-related burnout before and during COVID-19. PsyArXiv [preprint]. 10.31234/osf.io/vnkwa

[B41] Hochheimer C. Sabo R. Krist A. Day T. Cyrus J. Woolf S. (2016). Methods for evaluating respondent attrition in web-based surveys. J. Med. Internet Res. 18:e301. 10.2196/jmir.6342 27876687 PMC5141338

[B42] Holmberg K. Rosen D. Holland J. (1997). The Leisure Activities Finder, Self-Directed Search Form R, 2nd Edn. Odessa, FL: Psychological Assessment Resources.

[B43] Iob E. Steptoe A. Fancourt D. (2020). Abuse, self-harm and suicidal ideation in the UK during the COVID-19 pandemic. Br. J. Psychiatry. 2020:130. 10.1192/bjp.2020.130 32654678 PMC7360935

[B44] Jalloh M. Li W. Bunnell R. Ethier K. O'Leary A. Hageman K.. (2018). Impact of Ebola experiences and risk perceptions on mental health in Sierra Leone, July 2015. Br. Med. J. Glob. Health 3:e000471. 10.1136/bmjgh-2017-000471 29607096 PMC5873549

[B45] Kaufman J. Beghetto R. (2009). Beyond big and little: the four c model of creativity. Rev. Gen. Psychol. 13, 1–12. 10.1037/a0013688

[B46] King D. Delfabbro P. Billieux J. Potenza M. (2020). Problematic online gaming and the COVID-19 pandemic. J. Behav. Addict. 9, 184–186. 10.1556/2006.2020.00016 32352927 PMC8939428

[B47] Kiraly O. Potenza M. Stein D. King D. Hodgins D. Saunder J.. (2020). Preventing problematic internet use during the COVID-19 pandemic: CONSENSUS guidance. Comprehens. Psychiatry 100:152180. 10.1016/j.comppsych.2020.152180 32422427 PMC7215166

[B48] Ko C.-H. Yen J.-Y. (2020). Impact of COVID-19 on gaming disorder: monitoring and prevention. J. Behav. Addict. 9, 187–189. 10.1556/2006.2020.00040 32634111 PMC8939426

[B49] Kuykendall L. Boemerman L. Zhu Z. (2018). “The importance of leisure for subjective well-being,” in Handbook of Well-Being, eds Diener E. Oishi S. Tay L. Available online at: https://nobascholar.com/chapters/31/download.pdf (accessed June 08, 2021).

[B50] Kuykendall L. Tay L. Ng V. (2015). Leisure engagement and subjective well-being: a meta-analysis. Psychol. Bullet. 141, 364–403. 10.1037/a0038508 25602273

[B51] Lades L. Laffan K. Daly M. Delaney L. (2020). Daily emotional well-being during the COVID-19 pandemic. Br. J. Health Psychol. 2020:12450. 10.1111/bjhp.12450 32573074 PMC7361840

[B52] Lau J. Yang X. Tsui H. Pang E. Wing Y. (2006). Positive mental health-related impacts of the SARS epidemic on the general public in Hong Kong and their associations with other negative impacts. J. Infect. 53, 114–124. 10.1016/j.jinf.2005.10.019 16343636 PMC7132442

[B53] Leatherby L. Gelles D. (2020). How the Virus Transformed the Way Americans Spent Their Money. The New York Times. Available online at: https://www.nytimes.com/interactive/2020/04/11/business/economy/coronavirus-us-economy-spending.html (accessed June 08, 2021).

[B54] Lemieux T. Milligan K. Schirle T. Skuterud M. (2020). Initial impacts of the COVID-19 pandemic on the Canadian labour market. Can. Public Pol. 46, S55–S65. 10.3138/cpp.2020-049 PMC7977000 38629977

[B55] Lesser A. Nienhuis C. (2020). The impact of COVID-19 on physical activity behavior and well-being of Canadians. Int. J. Environ. Res. Publ. Health 17:3899. 10.3390/ijerph17113899 32486380 PMC7312579

[B56] Leversen I. Danielsen A. G. Birkeland M. S. Samdal O. (2012). Basic psychological need satisfaction in leisure activities and adolescents' life satisfaction. J. Youth Adolesc. 41, 1588–1599. 10.1007/s10964-012-9776-5 22627625 PMC3492701

[B57] Lufkin B. (2020). The Evolution of Home Fitness. British Broadcasting Corporation. Available online at: https://www.bbc.com/worklife/article/20200504-covid-19-update-quarantine-home-workouts-during-coronavirus (accessed June 08, 2021).

[B58] Matias T. Dominski F. Marks D. (2020). Human needs in COVID-19 isolation. J. Health Psychol. 25, 871–882. 10.1177/1359105320925149 32375564

[B59] Maugeri G. Castrogiovanni P. Battaglia G. Pippi R. D'Agata V. Palma A.. (2020). The impact of physical activity on psychological health during Covid-19 pandemic in Italy. Heliyon 6:e04315. 10.1016/j.heliyon.2020.e04315 32613133 PMC7311901

[B60] Mayo Clinic (2020). COVID-19 and Your Mental Health. Available online at: https://www.mayoclinic.org/mental-health-covid-19/art-20482731 (accessed June 08, 2021).

[B61] McNeely S. (2020). Order Amid Chaos: Why Jigsaw Puzzles Are a Popular Pandemic Pastime. Medical Xpress. Available online at: https://medicalxpress.com/news/2020-05-chaos-jigsaw-puzzles-popular-pandemic.html (accessed June 08, 2021).

[B62] Mestre-Bach G. Blycker G. Potenza M. (2020). Pornography use in the setting of the COVID-19 pandemic. J. Behav. Addict. 9, 181–183. 10.1556/2006.2020.00015 32663384 PMC8939420

[B63] Minsberg T. (2020). Running From Coronavirus: A Back-to-Basics Exercise Boom. The New York Times. Available online at: https://www.nytimes.com/2020/03/19/sports/running-exercise-coronavirus.html (accessed June 08, 2021).

[B64] Munford L. Fichera E. Sutton M. (2020). Is owning your home good for your health? Evidence from exogenous variations in subsidies in England. Econ. Hum. Biol. 39:100903. 10.1016/j.ehb.2020.100903 32659622 PMC7725589

[B65] Nakagawa S. Schielzeth H. (2012). A general and simple method for obtaining R2 from generalized linear mixed-effects models. Method. Ecol. Evol. 4, 144–152. 10.1111/j.2041-210x.2012.00261.x

[B66] Narici M. de Vito G. Franchi M. Paoli A. Moro T. Marcolin G.. (2020). Impact of sedentarism due to the COVID-19 home confinement on neuromuscular, cardiovascular and metabolic health: physiological and pathophysiological implications and recommendations for physical and nutritional countermeasures. Eur. J. Sport Sci. 2020:1761076. 10.1080/17461391.2020.1761076 32394816

[B67] Newman D. Tay L Diener E. (2014). Leisure and subjective well-being: a model of psychological mechanisms as mediating factors. J. Happiness Stud. 15, 555–578. 10.1007/s10902-013-9435-x

[B68] Office of National Statistics (2020). Coronavirus and How People Spent Their Time Under Lockdown: 28 March to 26 April 2020. Available online at: http://ons.gov.uk/economy/nationalaccounts/satelliteaccounts/bulletins/coronavirusandhowpeoplespenttheirtimeunderrestrictions/28marchto26april2020 (accessed June 08, 2021).

[B69] Paggi M. Jopp D. Hertzog C. (2016). The importance of leisure activities in the relationship between physical health and well-being in a life span sample. Gerontology 62, 450–458. 10.1159/000444415 26974682

[B70] Pecanha T. Goessler K. Roschel H. Gualano B. (2020). Social isolation during the COVID-19 pandemic can increase physical inactivity and the global burden of cardiovascular disease. Am. J. Physiol. Heart Circulat. Physiol. 318, H1441–H1446. 10.1152/ajpheart.00268.2020 32412779 PMC7303725

[B71] Public Health Agency (2020). Rediscovering Home Cooking – A Positive Outcome From the COVID-19 Lockdown. Available online at: https://www.publichealth.hscni.net/node/5167 (accessed June 08, 2021).

[B72] Qiu J. Shen B. Zhao M. Wang Z. Xie B. Xu Y. (2020). A nationwide survey of psychological distress among Chinese people in the COVID-19 epidemic: implications and policy recommendations. Gen. Psychiatry 33:e100213. 10.1136/gpsych-2020-100213 32215365 PMC7061893

[B73] R Core Team (2020). R: A Language and Environment for Statistical Computing. R Foundation for Statistical Computing. Available online at: http://www.r-project.org/index.html (accessed June 08, 2021).

[B74] Rajkumar R. (2020). COVID-19 and mental health: a review of the existing literature. Asian J. Psychiatry 52:102066. 10.1016/j.ajp.2020.102066 32302935 PMC7151415

[B75] Revelle R. (2017). Psych: Procedures for Psychological, Psychometric, and Personality Research. Comprehensive R Archive Network. Available online at: https://CRAN.R-project.org/package=psych (accessed June 08, 2021).

[B76] Rodriguez-Rey R. Garrido-Hernansaiz H. Collado S. (2020). Psychological impact and associated factors during the initial stage of the coronavirus (COVID-19) pandemic among the general population in Spain. Front. Psychol. 11:1540. 10.3389/fpsyg.2020.01540 32655463 PMC7325630

[B77] Ryu J. Heo J. (2018). Relationships between leisure activity types and well-being in older adults. Leisure Stud. 37, 331–342. 10.1080/02614367.2017.1370007

[B78] Schimmack U. (2008). “The structure of subjective well-being,” in The Science of Subjective Well-Being, eds Eid M. Larsen R. (Guilford Press), 97–123. Available online at: http://citeseerx.ist.psu.edu/viewdoc/download?doi=10.1.1.320.2222&rep=rep1&type=pdf (accessed June 08, 2021).

[B79] Sen-Crowe B. McKenney M. Elkbuli A. (2020). Social distancing during the COVID-19 pandemic: staying home save lives. Am. J. Emerg. Med. 38, 1519–1520. 10.1016/j.ajem.2020.03.063 32305155 PMC7194642

[B80] Slimani M. Paravlic A. Mbarek F. Bragazzi N. Tod D. (2020). The relationship between physical activity and quality of life during the confinement induced by COVID-19 outbreak: a pilot study in Tunisia. Front. Psychol. 11:1882. 10.3389/fpsyg.2020.01882 32849104 PMC7427614

[B81] Smith L. Jacob L. Butler L. Schuch F. Barnett Y. Grabovac I.. (2020). Prevalence and correlates of physical activity in a sample of UK adults observing social distancing during the COVID-19 pandemic. Br. Med. J. Open Sport Exerc. Med. 6:e000850. 10.1136/bmjsem-2020-000850 PMC7358093 34192006

[B82] Sport England (2020). New Toolkit Launched to Boost Physical Activity Despite Coronavirus. Available online at: https://www.sportengland.org/news/new-toolkit-launched-boost-physical-activity-despite-coronavirus (accessed June 08, 2021).

[B83] Stuckey H. Nobel J. (2010). The connection between art, healing, and public health: a review of current literature. Am. J. Public Health 100, 254–263. 10.2105/AJPH.2008.156497 20019311 PMC2804629

[B84] Szumilas M. (2010). Explaining odds ratios. J. Can. Acad. Child Adolesc. Psychiatry 19, 227–229. 20842279 PMC2938757

[B85] Teclaw R. Price M. C. Osatuke K. (2012) Demographic question placement: effect on item response rates means of a veterans' health administration survey. J. Bus. Psychol. 27, 281–290. 10.1007/s10869-011-9249-y

[B86] ten Berge J. Krijnen W. Wansbeek T. Shapiro A. (1999). Some new results on correlation-preserving factor scores prediction methods. Linear Algebra Appl. 289, 311–318. 10.1016/S0024-3795(97)10007-6

[B87] The National Health Service (2020). Mental Wellbeing While Staying at Home. Available online at: https://www.nhs.uk/oneyou/every-mind-matters/coronavirus-covid-19-staying-at-home-tips/ (accessed June 08, 2021).

[B88] Topp C. W. Ostergaard S. D. Sondergaard S. Bech P. (2015). The WHO-5 well-being index: a systematic review of the literature. Psychother. Psychosomat. 84, 167–176. 10.1159/000376585 25831962

[B89] Torales J. O'Higgins M. Castaldelli-Maia J. Ventriglio A. (2020). The outbreak of COVID-19 coronavirus and its impact on global mental health. Int. J. Soc. Psychiatry 66, 317–320. 10.1177/0020764020915212 32233719

[B90] Tull M. Edmonds K. Scamaldo K. Richmond J. Rose J. Gratz K. (2020). Psychological outcomes associated with stay-at-home orders and the perceived impact of COVID-19 on daily life. Psychiatry Res. 289:113098. 10.1016/j.psychres.2020.113098 32434092 PMC7252159

[B91] United Kingdom Cabinet Office (2021). (COVID-19) Coronavirus Restrictions: What You Can and Cannot Do. Available online at: https://www.gov.uk/guidance/covid-19-coronavirus-restrictions-what-you-can-and-cannot-do (accessed June 08, 2021).

[B92] Viana R. de Lira C. (2020). Exergames as coping strategies for anxiety disorders during the COVID-19 quarantine period. Games Health J. 9, 147–149. 10.1089/g4h.2020.0060 32375011

[B93] Wheatley D. Bickerton C. (2019). Measuring changes in subjective well-being from engagement in the arts, culture and sport. J. Cult. Econ. 43, 421–442. 10.1007/s10824-019-09342-7

[B94] Wiese C. W. Kuykendall L. Tay L. (2018). Get active? A meta-analysis of leisure-time physical activity and subjective well-being. J. Posit. Psychol. 13, 57–66. 10.1080/17439760.2017.1374436

[B95] Williams S. Armitage C. Tampe T. Dienes K. (2020). Public perceptions and experiences of social distancing and social isolation during the COVID-19 pandemic: a UK-based focus group study. Br. Med. J. Open 10:e039332. 10.1101/2020.04.10.20061267 32690752 PMC7387310

[B96] Willis E. (2020). Coronavirus: How Artists Are Depicting the Lockdown. British Broadcasting Corporation News. Available online at: https://www.bbc.co.uk/news/uk-52296886 (accessed June 08, 2021).

[B97] Wong M. (2020). Stanford Research Provides a Snapshot of a New Working-From-Home Economy. Stanford News. Available online at: https://news.stanford.edu/2020/06/29/snapshot-new-working-homeeconomy/ (accessed June 08, 2021).

[B98] World Health Organization (2020a). #HealthyAtHome - Mental Health: Looking After Our Mental Health. Available online at: https://www.who.int/campaigns/connecting-the-world-to-combat-coronavirus/healthyathome/healthyathome—mental-health(accessed June 08, 2021).

[B99] World Health Organization (2020b). Physical Activity. Available online at: https://www.who.int/news-room/fact-sheets/detail/physical-activity (accessed June 08, 2021).

[B100] Yang J. Suh C. Lee C. Son B. (2018). The work-life balance and psychosocial well-being of South Korean workers. Ann. Occup. Environ. Med. 30:38. 10.1186/s40557-018-0250-z 29928507 PMC5989347

