# Supplementary material for: Creativity and Leisure During COVID-19: Examining the Relationship Between Leisure Activities, Motivations, and Psychological Well-Being
Source: Front Psychol. 2021 Jul 5;12:609967. doi: 10.3389/fpsyg.2021.609967 (PMC8288551; doi:10.3389/fpsyg.2021.609967)
Supplement: Supplementary file 1 [file Table_1.docx]

| Supplemental Table 1 | |  |  |  |  |  |  |
| --- | --- | --- | --- | --- | --- | --- | --- |
| WHO-5 well-being coefficient estimates | |  |  |  |  |  |  |
|  | Estimate | | Std. Error | t value | P-value | 95% CI lower | 95% CI upper |
| Intercept | 40.74 | | 1.48 | 27.49 | <.001 | 37.84 | 43.65 |
| Age (mean-centered) | 0.31 | | 0.03 | 9.52 | <.001 | 0.25 | 0.38 |
| Gender: Female | -6.89 | | 0.69 | -9.94 | <.001 | -8.25 | -5.53 |
| Gender: Non-binary | -16.18 | | 2.19 | -7.40 | <.001 | -20.47 | -11.89 |
| Gender: Other | -13.88 | | 9.08 | -1.53 | 0.126 | -31.69 | 3.92 |
| Gender: Undisclosed | -8.23 | | 3.68 | -2.24 | 0.025 | -15.44 | -1.02 |
| Education: Doctorate | -1.84 | | 1.24 | -1.49 | 0.137 | -4.27 | 0.59 |
| Education: High school | 1.80 | | 0.93 | 1.94 | 0.053 | -0.02 | 3.62 |
| Education: Master's degree | 0.62 | | 0.85 | 0.73 | 0.465 | -1.04 | 2.28 |
| Education: Other | -1.71 | | 1.87 | -0.91 | 0.360 | -5.37 | 1.95 |
| Education: Vocational training | 0.78 | | 1.08 | 0.73 | 0.468 | -1.33 | 2.90 |
| Employment: Paid leave | 1.05 | | 1.38 | 0.76 | 0.447 | -1.66 | 3.76 |
| Employment: Physically attending work | -0.31 | | 0.99 | -0.31 | 0.758 | -2.25 | 1.64 |
| Employment: Other | -2.99 | | 1.63 | -1.84 | 0.066 | -6.18 | 0.19 |
| Employment: Retired | 1.71 | | 1.81 | 0.94 | 0.345 | -1.84 | 5.26 |
| Employment: Stay-at-home parent | -3.47 | | 1.78 | -1.94 | 0.052 | -6.97 | 0.03 |
| Employment: Student | 0.51 | | 1.06 | 0.48 | 0.632 | -1.57 | 2.59 |
| Employment: Unemployed, no financial compensation | -4.20 | | 1.25 | -3.35 | 0.001 | -6.65 | -1.74 |
| Employed: Unemployed with financial compensation | 0.13 | | 1.28 | 0.10 | 0.918 | -2.38 | 2.64 |
| Household number | 0.42 | | 0.24 | 1.75 | 0.081 | -0.05 | 0.90 |
| COVID-19 vulnerability: Don't know | -0.36 | | 1.41 | -0.26 | 0.797 | -3.14 | 2.41 |
| COVID-19 vulnerability: No | 3.60 | | 0.67 | 5.37 | <.001 | 2.29 | 4.92 |
| Social distancing: No | -1.81 | | 2.22 | -0.82 | 0.415 | -6.16 | 2.54 |
| Social distancing: Yes, some restrictions lifted | 0.11 | | 0.67 | 0.16 | 0.874 | -1.21 | 1.43 |
| Social distancing: Yes, all restrictions lifted | 2.14 | | 2.28 | 0.94 | 0.347 | -2.32 | 6.60 |
| Leisure activity engagement: Same amount of time | 9.77 | | 1.07 | 9.13 | <.001 | 7.67 | 11.87 |
| Leisure activity engagement: More time | 12.08 | | 0.92 | 13.19 | <.001 | 10.28 | 13.88 |
| New activities: Yes | 0.81 | | 0.69 | 1.19 | 0.235 | -0.53 | 2.16 |
| Note: Coefficients are expressed as WHO-5 scores from 0-100. Reference levels are gender= male; education= university degree; employment status= working from home; COVID-19 vulnerability= no; social distancing= yes, currently; leisure time= no, new activities= no | | | | | | | |
