# Supplementary material for: Creativity and Leisure During COVID-19: Examining the Relationship Between Leisure Activities, Motivations, and Psychological Well-Being
Source: Front Psychol. 2021 Jul 5;12:609967. doi: 10.3389/fpsyg.2021.609967 (PMC8288551; doi:10.3389/fpsyg.2021.609967)
Supplement: Supplementary file 2 [file Table_2.docx]

| Supplemental Table 2 | | |
| --- | --- | --- |
| Activity Category Frequency | | |
| Activity Category | Activity | Frequency |
| Sport and outdoor pursuits | Walking | 857 |
|  | Watching sport | 764 |
|  | Running | 598 |
|  | Trekking/Hiking | 465 |
|  | Cycling | 416 |
|  | Keeping fit | 392 |
|  | Gym | 319 |
|  | Yoga | 245 |
|  | Swimming | 237 |
|  | Outdoor pursuit | 161 |
|  | Weightlifting | 154 |
|  | Rollerblading/Skating/Skateboarding | 133 |
|  | Dog walking | 130 |
|  | Climbing | 118 |
|  | Tennis/Badminton/Squash | 76 |
|  | Camping | 70 |
|  | Basketball/Ball sport | 68 |
|  | Canoe/Kayak/Row/Surf | 68 |
|  | Sport | 68 |
|  | Archery/Shooting | 67 |
|  | Tai Chi/Judo | 64 |
|  | Football | 63 |
|  | Athletics | 60 |
|  | Orienteering | 57 |
|  | Pilates/Aerobic classes | 57 |
|  | Frisbee/Softball | 45 |
|  | Fishing | 42 |
|  | Golf | 36 |
|  | Motor sport | 36 |
|  | Horse riding | 33 |
|  | Mountaineering/Bikes | 32 |
|  | Sailing/Boating | 32 |
|  | Skiing/Snowboarding | 30 |
|  | Slacklining/Gymnastics/Balance | 24 |
|  | Hockey | 21 |
|  | Bowls/Skittles/Croquet | 16 |
|  | Rugby | 13 |
|  | Scuba, diving | 11 |
|  | Boxing | 10 |
|  | Billiards/Snooker | 9 |
|  | Cricket/(Baseball) | 9 |
|  | Table Tennis | 9 |
|  | Fell/Long Distance Running | 8 |
|  | Flying | 4 |
|  | Paintball | 3 |
|  | Gliding and Skydiving | 2 |
|  | Wrestling | 1 |
| Musical and performing arts engagement | Playing instrument | 888 |
|  | DJing/Sound producing | 270 |
|  | Dancing | 184 |
|  | Singing | 99 |
|  | Writing music | 64 |
|  | Juggling/Circus | 31 |
|  | Performing theatre | 21 |
|  | Acting | 15 |
|  | Writing songs | 13 |
|  | Unicycling | 4 |
|  | Piano tuning | 1 |
| Niche and IT interests | Programming | 135 |
|  | Birdwatching/Wildlife | 82 |
|  | Aquarium/amphibian keeping | 65 |
|  | Yoyo/Technical Game | 57 |
|  | Darkroom/3D printing | 52 |
|  | Electronics/Engineering | 52 |
|  | Family history | 41 |
|  | Mechanics | 29 |
|  | Mathematics | 18 |
|  | Collecting | 17 |
|  | Computing | 15 |
|  | Business | 14 |
|  | Biology/Biomedical sciences | 11 |
|  | Astronomy | 10 |
|  | Collecting (coins, stamps, metal) | 10 |
|  | Historical reenactment/Cosplay | 10 |
|  | Botany/Ecology | 9 |
|  | History | 8 |
|  | Magic | 6 |
|  | Vehicle spotting/Appreciation | 6 |
|  | Naturopathic medicine | 4 |
|  | Bird keeping | 3 |
|  | Hermeneutics/Religious Studies | 3 |
|  | Pens | 3 |
|  | Psychology | 3 |
|  | Rockhounding | 3 |
|  | Antiques | 2 |
|  | Archaeology | 2 |
|  | Classical studies | 2 |
|  | Entomology | 2 |
|  | Mycology | 2 |
|  | Sports (research) | 2 |
|  | Taxidermy | 2 |
|  | Bellringing | 1 |
|  | Heraldry | 1 |
|  | Horology | 1 |
|  | Linguistics | 1 |
|  | Mythological studies | 1 |
|  | Ornithology | 1 |
|  | Signs | 1 |
| Creative consumption | Listening to music | 1507 |
|  | Film-going | 875 |
|  | Concerts | 505 |
|  | Musical activity unspecified | 229 |
|  | Theatre | 215 |
|  | Museums/Library | 85 |
|  | Art Galleries | 31 |
|  | Watching comedy | 17 |
|  | Opera | 10 |
|  | Ballet | 9 |
| Fine arts | Painting | 266 |
|  | Drawing | 242 |
|  | Photography | 96 |
|  | Papercraft | 57 |
|  | Animation/Video producing | 37 |
|  | Glassmaking/Stained glass | 34 |
|  | Calligraphy | 32 |
|  | Website/graphic design | 25 |
|  | Sculpture/Clay | 20 |
|  | Pottery/Ceramics | 18 |
|  | Acrobatic flying | 6 |
|  | Mosaic | 3 |
| Home crafts and artisanship | Knitting/Crochet | 1217 |
|  | Embroidery/Needlework | 806 |
|  | Cooking | 651 |
|  | Gardening | 622 |
|  | Baking | 618 |
|  | DIY | 204 |
|  | Crafts | 151 |
|  | Breadmaking | 137 |
|  | Model making | 107 |
|  | Woodworking | 101 |
|  | Domestic Chores | 96 |
|  | Making alcohol/Fermented food | 86 |
|  | Houseplant gardening | 66 |
|  | Basketry/Weaving | 62 |
|  | Scrapbooking/Bullet Journal | 48 |
|  | Coloring | 44 |
|  | Culture | 39 |
|  | Home decorating/House painting | 39 |
|  | Jewelry making | 38 |
|  | Carving and Carpentry | 33 |
|  | Card making/Printmaking | 32 |
|  | Doll/miniature making | 29 |
|  | Leatherworking | 29 |
|  | Cake decorating | 25 |
|  | Nail art/Makeup | 22 |
|  | Farming | 21 |
|  | Beading | 20 |
|  | Growing vegetables | 19 |
|  | Metalworking | 19 |
|  | Foraging | 16 |
|  | Bookbinding | 15 |
|  | Furniture/Instrument making | 15 |
|  | Soapmaking/Candlemaking | 14 |
|  | Spinning/Textile | 14 |
|  | Flower arranging/Wreaths | 10 |
|  | Macrame/String art | 9 |
|  | Beekeeping/Antkeeping | 7 |
|  | Dollhouse construction/Diorama | 7 |
|  | Upholstering | 7 |
|  | Collage/Decoupage | 6 |
|  | Dyeing | 6 |
|  | Rug making | 6 |
|  | Cartography/mapmaking | 4 |
|  | Hydroponics | 4 |
|  | Welding | 4 |
|  | Architecture | 3 |
|  | Miniatures (unspecified) | 2 |
|  | Blacksmithing | 1 |
|  | Bonsai | 1 |
| Mind games | Strategic Computer | 982 |
|  | Jigsaw puzzles | 200 |
|  | Board Games | 162 |
|  | Computer Games (unspecified) | 121 |
|  | Role-play Games | 94 |
|  | Crosswords | 41 |
|  | Pub Quiz | 39 |
|  | Chess | 25 |
|  | Social/Competitive Cards | 20 |
|  | Coded Puzzles | 12 |
|  | Sudoku | 8 |
|  | Rubiks/Spatial | 6 |
|  | Solo Card Games | 4 |
|  | Logic Puzzles | 3 |
|  | Picture Logic puzzles | 2 |
|  | Scrabble | 2 |
|  | Word Games | 1 |
| Public service/leadership | Volunteering | 105 |
|  | Instructing | 52 |
|  | Church activities | 38 |
|  | Coaching | 24 |
|  | Politics | 17 |
|  | Scouting | 16 |
|  | Club organising | 14 |
|  | Journalism/broadcasting | 5 |
|  | Public speaking/Debate | 3 |
|  | Preaching | 2 |
| Reading, writing and languages | Reading | 1689 |
|  | Languages | 581 |
|  | Writing | 215 |
|  | Letter writing/Journaling | 102 |
|  | Blogging | 16 |
|  | Book Club | 10 |
|  | Editing | 3 |
|  | Translating | 3 |
|  | Literature | 1 |
| Social, food and relaxation | Watching TV/Netflix/Movie | 3410 |
|  | Audiobook/Podcast/Radio | 720 |
|  | Eating out | 440 |
|  | Socialising | 416 |
|  | Internet/Social Media | 380 |
|  | Friends | 351 |
|  | Shopping/Car Boot Sales | 274 |
|  | Family | 236 |
|  | Mindfulness/Meditation/Self-care | 136 |
|  | Online chatting/Video call | 67 |
|  | Alcohol | 64 |
|  | Sleeping/Resting | 56 |
|  | Social clubs | 42 |
|  | People watching | 33 |
|  | Pets | 30 |
|  | Zoo/Amusement/Aquarium | 25 |
|  | Sex | 23 |
|  | Drugs | 23 |
|  | Planning/Organizing | 22 |
|  | Barbeque | 17 |
|  | Online shopping | 14 |
|  | Dog training | 12 |
|  | Youth organization | 12 |
|  | Flea market | 11 |
|  | Coffee/tea making | 10 |
|  | Stocks and shares | 9 |
|  | Picnicking | 6 |
|  | Property | 5 |
|  | Gambling | 4 |
|  | Conservation organization | 3 |
|  | Escape room | 3 |
|  | Self-help groups | 3 |
|  | Tobacco | 3 |
| Travel | Travel | 348 |
|  | Driving | 49 |
| Work/study unspecified | Research/Study/University | 286 |
|  | Work/Entrepreneur | 120 |
