# Supplementary material for: Creativity and Leisure During COVID-19: Examining the Relationship Between Leisure Activities, Motivations, and Psychological Well-Being
Source: Front Psychol. 2021 Jul 5;12:609967. doi: 10.3389/fpsyg.2021.609967 (PMC8288551; doi:10.3389/fpsyg.2021.609967)
Supplement: Supplementary file 3 [file Data_Sheet_1.docx]

Questionnaire Text

1. What is your age?

2. What gender do you identify as?

-Male

-Female

-Non-binary

-Other

-Prefer not to say

3. What is the highest level of education you have completed or are currently completing?

-High school/secondary school equivalent

-Vocational/professional training

-University degree

-Master’s degree

-Doctorate

-Other (please specify)

4. What country do you currently live in?

5. Have you had to stay at home or practice other social distancing measures severely restricting your movements because of COVID-19?

-Yes, currently

-Yes, currently, but some restrictions have been lifted

-Yes, previously, but all restrictions have been lifted

-No

6. Please indicate for each of the 5 statements which is closest to how you have been feeling over the past 2 weeks.

Options: At no time, Some of the time, Less than half of the time, More than half of the time, Most of the time, All of the time

-I have felt cheerful and in good spirits

-I have felt calm and relaxed

-I have felt active and vigorous

-I woke up feeling fresh and rested

-My daily life has been filled with things that interest me

7. Compared to this time last year, I feel that time is

-1 (Really dragging)

-2

-3 (Passing no differently)

-4

-5 (Speeding by)

8. For this section, think about the leisure activities you engaged in during a typical month last year (that is, before COVID-19). We consider an activity any pastime you pursued or participated in because you wanted to. This might be a hobby, interest, or anything else you did during your spare time.

You can list up to a maximum of 5 active and 5 passive activities. If you can't think of that many, you can leave the extra spaces blank.

ACTIVE = You are making or doing something, like playing sports, learning languages, or woodworking

[space for 1-5]

PASSIVE = You are a spectator, like watching sports, listening to music, or going to the movies.

[space for 1-5]

9. Please indicate whether you have spent more, less, or the same amount of time on these activities during the time your area has been affected by COVID-19 (e.g., in the past 2 months).

If you listed fewer than 10 activities, you will see some empty spaces. Please leave these blank

Options: Less time, same amount of time, more time [each activity carried through]

10. In addition to the activities above, have you taken up any new or rediscovered any old activities that you used to do?

-Yes

-No

11. Please list any additional activities you have engaged in during the time your area has been affected by COVID-19. You should also indicate whether each activity is completely new to you or if it is a rediscovered activity that you used to do.

You can list up to a maximum of 3 active and 3 passive activities. If you can't think of that many, you can leave the extra spaces blank.

ACTIVE = You are making or doing something, like playing sports, learning languages, or woodworking.

PASSIVE = You are a spectator, like watching sports, listening to music, or going to the movies

[for each] This activity is [new] or [rediscovered]

12. In general, compared to this time last year, I am spending

-More time overall on leisure activities

-Less time overall on leisure activities

-About the same amount of time overall

13. Now think about why you engaged in any of these activities during COVID-19 (e.g., in the past 2 months) and rate the importance of the following reasons. Your ratings don't have to reflect your feelings about all of the activities you listed. For example, if "to keep in shape" only applies to one activity, you can still rate it as high or as low as you would like. If you aren't sure, just go with your gut feeling.

Options: 1 (Not at all), 2, 3 (Neutral), 4, 5 (Very much so)

-To change my daily routine

-To get away from the responsibilities of everyday life

-To exercise or keep in shape

-To get away from civilization for a while

-To be close to nature

-To be creative

-To relax physically

-To let my mind slow down for a while

-To keep contact with family or friends

-To get away from other people

-To talk to new and varied people

-To help bring the family together more

-To show others I could do something

-To make others think highly of me

-To be in charge or run things

-To help others

-For the excitement

-To see the results of my efforts

-To practice my skills and abilities

-Because of the competition

-To learn what I am capable of

-To keep busy

-To avoid boredom

-To use my mind

-To think about my personal values

-To help focus better on my work

-To feel as though I am in control of something in my life

- To improve my mood

-To manage my stress

-To give me a sense of purpose

-To be good at something

-To feel that I am nurturing or taking care of something

-To get away from the news about current events

14. If you have generally been spending less time on leisure activities during COVID-19, please rate the extent to which the following reasons have influenced this:

Options: 1 (Not at all), 2, 3 (Neutral), 4, 5 (Very much so)

-I am busy working from home

-I am busy working outside the home

-I have childcare responsibilities

-I have caring responsibilities (excluding childcare)

-I am busy with school or university

-I have experienced problems with my physical health

-I do not have adequate transportation

-The place(s) I would go for my activities are closed

-I don't have anyone to do them with

-I am experiencing financial difficulties

-I do not have adequate resources

-I have been feeling less motivated

-I have been feeling low or depressed

-I have been feeling anxious

-I have had difficulty concentrating

-I have less free time available

-Other (please specify):

15. Do you intend to keep doing any of these activities after COVID-19?

If you listed fewer than 16 activities, you will see some empty spaces. Please leave these blank

Options: 1 (I do not plan to continue this activity), 2, 3 (Not sure), 4, 5 (I definitely plan to continue this activity)

[for each activity]

16. Almost done! How many people currently live in your household?

17. With whom have you been living during COVID-19? Check all that apply.

-Housemates or friends

-My partner

-My children

-My parents or other family members (siblings, grandparents, etc.)

-My pets

-Alone

-Other (please specify)

18. Is anyone in your household considered vulnerable to COVID-19?

-Yes

-No

-Don’t know or decline to answer

19. Which of the following best describes your employment status during COVID-19?

-Employed, physically attending my place of work

-Employed, working from home

-Employed on paid leave

-Unemployed, receiving financial compensation

-Unemployed, not receiving financial compensation

-Stay-at-home parent

-Retired

-Student

-Other

20. What is or was your main paid occupation? Your answer should focus on your field or area of work, rather than the level of appointment held.

If you are unemployed or retired, please indicate your most recent occupation.

If you are a student, teacher, or lecturer, please also include your subject area
